# The wound inflammatory response exacerbates growth of pre-neoplastic cells and progression to cancer

**DOI:** 10.15252/embj.201490147

**Published:** 2015-07-01

**Authors:** Nicole Antonio, Marie Louise Bønnelykke-Behrndtz, Laura Chloe Ward, John Collin, Ib Jarle Christensen, Torben Steiniche, Henrik Schmidt, Yi Feng, Paul Martin

**Affiliations:** 1 School of Biochemistry, University of BristolBristol, UK; 2 Department of Experimental Clinical Oncology, Aarhus UniversityAarhus, Denmark; 3 Department of Plastic and Reconstructive Surgery, Aarhus UniversityAarhus, Denmark; 4 School of Physiology and Pharmacology, University of BristolBristol, UK; 5 Department of Public Health, University of CopenhagenCopenhagen, Denmark; 6 Department of Pathology, Aarhus UniversityAarhus, Denmark; 7 Department of Clinical Medicine, Aarhus UniversityAarhus, Denmark; 8 Department of Oncology, Aarhus UniversityAarhus, Denmark; 9 MRC Centre for Inflammation Research, University of EdinburghEdinburgh, UK; 10 School of Medicine, University of CardiffCardiff, UK

**Keywords:** cancer inflammation, cancer surgery, live imaging, melanoma, wound healing

## Abstract

There is a long-standing association between wound healing and cancer, with cancer often described as a “wound that does not heal”. However, little is known about how wounding, such as following surgery, biopsy collection or ulceration, might impact on cancer progression. Here, we use a translucent zebrafish larval model of Ras^G12V^-driven neoplasia to image the interactions between inflammatory cells drawn to a wound, and to adjacent pre-neoplastic cells. We show that neutrophils are rapidly diverted from a wound to pre-neoplastic cells and these interactions lead to increased proliferation of the pre-neoplastic cells. One of the wound-inflammation-induced trophic signals is prostaglandin E_2_ (PGE_2_). In an adult model of chronic wounding in zebrafish, we show that repeated wounding with subsequent inflammation leads to a greater incidence of local melanoma formation. Our zebrafish studies led us to investigate the innate immune cell associations in ulcerated melanomas in human patients. We find a strong correlation between neutrophil presence at sites of melanoma ulceration and cell proliferation at these sites, which is associated with poor prognostic outcome.

See also: **SK Wculek & I Malanchi** (September 2015)

## Introduction

Chronic, persistent inflammation has been shown to damage affected organs and may predispose to many diseases ranging from diabetes to cancer (O’Byrne & Dalgleish, 2001). Epidemiological studies indicate that at least 20% of all cancers begin as a direct consequence of chronic inflammatory disease in different tissues and organs (Grivennikov *et al*, 2010), and inflammation is considered to be one of the ten “hallmarks of cancer” (Hanahan & Weinberg, 2011). Inflammation-driven cancers include those associated with chronic viral infections such as hepatitis and hepatocellular carcinoma (Lin *et al*, 2015) or bacterial infections such as *Helicobacter pylori*, which accounts for almost all stomach cancers (Mantovani & Sica, 2010). Similarly, local chronic inflammation is a consequence of attack by the parasitic worm *Schistosoma hematobium* causing bladder cancer in some parts of the world (Condeelis & Pollard, 2006). Local chronic tissue inflammation also often leads to malignant transformation (Werner & Schafer, 2008), as for example in Barrett’s oesophagus (Colleypriest *et al*, 2009). Moreover, studies have shown that patients receiving long-term therapy (>5 years) with anti-inflammatory drugs, such as aspirin, have fewer relapses or appearances of new tumours, particularly colon cancer, providing further evidence for the pro-cancer influence of long-term inflammation (Rothwell *et al*, 2011).

Surgery is a key cancer therapy and is still the most effective means to treat human solid cancers, which have not yet metastasised (Ceelen *et al*, 2013). However, tissue damage is implicated as a possible trigger in the development of various cancers (Combemale *et al*, 2007; Lee *et al*, 2009; Kasper *et al*, 2011; Senet *et al*, 2012) and may provide a favourable niche for tumour reoccurrence (Hofer *et al*, 1998), as well as facilitating the growth of pre-existing micro-metastases (Bogden *et al*, 1997), suggesting that surgery may have clinical consequences beyond simply removing the primary cancer (Kuraishy *et al*, 2011; O’Leary *et al*, 2013). Classic studies have shown that wounding can lead to tumourigenesis (Hennings & Boutwell, 1970; Clark-Lewis & Murray, 1978; Leder *et al*, 1990); for example, wounding Rous sarcoma virus-infected chickens led to 100% tumour formation at the injured site (Sieweke *et al*, 1990), and transgenic mice carrying the *v-jun* oncogene developed dermal fibrosarcomas after full thickness wounding, whereas identical wounds in non-transgenic mice healed without tumour formation (Schuh *et al*, 1990). A more recent retrospective analysis suggested that the use of anti-inflammatories such as ketorolac (a non-steroidal anti-inflammatory drug) given to patients before and after mastectomy led to a lower reoccurrence of their breast cancer, implicating surgery-mediated inflammation as a key initiator of wound-induced cancer growth (Forget *et al*, 2010; Retsky *et al*, 2013).

Under normal acute inflammatory situations, such as after tissue damage, or an infection, the inflammatory response is self-limiting and immune cells resolve by apoptosis or returning to the circulation (Martin & Shaw, 2009). In malignant tissues, however, pro-inflammatory signals continue to intensify to support the needs of the tumour. Hence, the inflammatory response never resolves, and tumours have been likened to “wounds that do not heal” (Dvorak, 1986; Chang *et al*, 2004; Werner & Schafer, 2008; Troester *et al*, 2009). Tumour-associated macrophages (TAMs) and neutrophils (TANs) can constitute a large proportion of the tumour mass (Condeelis & Pollard, 2006) and are associated with poor prognosis in human patients, particularly when tumour-derived cytokines induce macrophage differentiation from a tumouricidal M1 phenotype to an M2 phenotype, which favours growth and tissue remodelling (Biswas *et al*, 2006).

In this study, we have utilised zebrafish as a model organism to visualise the relationship between wound-associated inflammation and adjacent cancers as they develop *in vivo*. The translucency of zebrafish larvae enables us to live image these interactions from the earliest stages when a pre-neoplastic cell first arises in otherwise healthy tissue. We express oncogenic Ras^G12V^ in specific cell types: melanocytes (to model melanoma) and goblet cells (modelling rare carcinoid tumours). We have previously shown that neutrophils and macrophages interact with the pre-neoplastic cells, even at a single-cell stage, before these cells divide to form clones (Feng *et al*, 2010), and that these interactions are beneficial for pre-neoplastic cell growth, in part due to release of trophic factors such as PGE_2_ from myeloid cells (Feng *et al*, 2012). Here, we show that immune cells recruited to a wound are rapidly drawn out from the wound by competing signals from pre-neoplastic cells. The amplified exposure to innate immune cells is associated with increased proliferation of pre-neoplastic cells that we show is dependent upon innate immune cells. We go on to extrapolate these mechanistic studies in zebrafish to human, clinical samples of melanomas with superimposed wounds (ulceration), and we find a close correlation of neutrophil influx with proliferative index of cancer cells in these ulcerated melanomas. We have previously seen that the presence of tumour infiltrating neutrophils and macrophages in primary melanomas was correlated with poor survival (Jensen *et al*, 2009, 2012). We now show that neutrophil number correlates with proliferation from non-ulcerated melanomas through to moderate ulceration, and this proliferative microenvironment may help to explain why wound-induced inflammation may be detrimental to patient survival.

## Results

### Adult zebrafish tumours arise at sites of repeated tissue wounding

**Figure 1:**
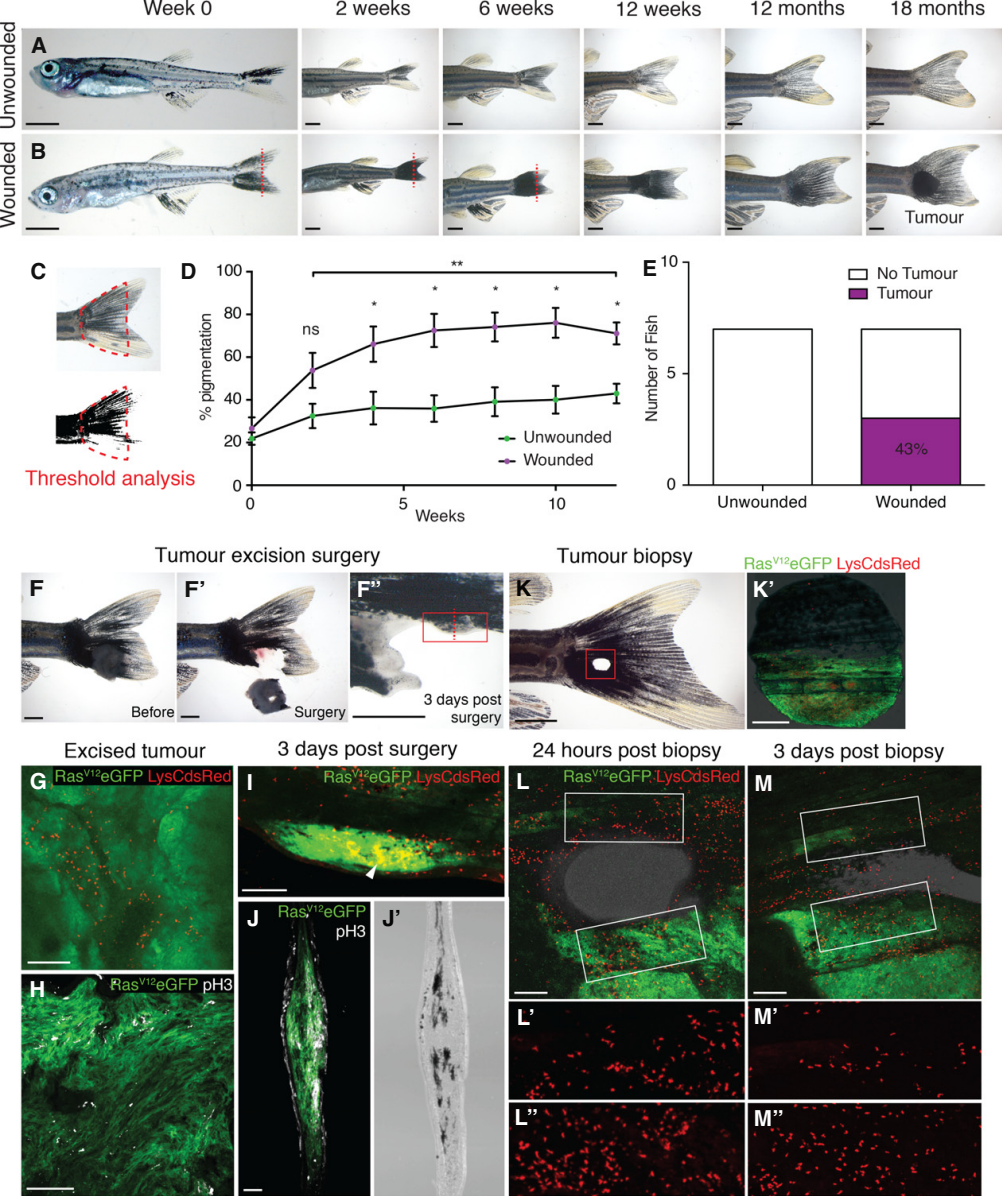
Tumours arise at sites of injury A Unwounded 4-week-old *kita*:Ras^G12V^eGFP juvenile fish followed over time.
B Juvenile *kita*:Ras^G12V^eGFP fish wounded by tail-fin resection (red line) every 2 weeks for 12 weeks.
C Pigmentation of the tail fin as quantified by threshold analysis of the tail area (indicated by red dotted line) at each time point.
D Graph illustrating the percent pigmentation over 12 weeks (mean ± SEM).
E Number of tail fin tumours developed in wounded versus unwounded fish (*n* = 7 fish in unwounded and wounded group, respectively).
F–F’’ An adult *kita*:Ras^G12V^eGFP fish with a large tail melanoma prior to surgery (F), immediately after (F’) and 3 days post-surgery (F’’); the surgery left a small section of melanoma remaining on the ventral tail fin (highlighted by the red box).
G Whole-mount immunohistochemistry of the excised unwounded tumour stained for LysC (red) to reveal neutrophils.
H Immunohistochemistry of a section of the same excised tumour stained for phospho-histone H3 (pH3, white) to reveal the extent of proliferation.
I Whole-mount immunohistochemistry of the tail with wounded tumour remnant 3 days post-surgery (green) illustrating accumulation of neutrophils (red) within the wounded tumour tissue (arrowhead).
J, J’ A frozen section through the tail with wounded tumour remnant 3 days post-surgery (plane indicated by red dotted line in F’’) stained for phospho-H3 revealing how proliferating cells (white) appear to have increased in number in the remaining tumour. Equivalent bright-field section shown in (J’).
K, K’ An adult *kita*:Ras^G12V^eGFP; p53^+/−^; LysC:dsRed fish with an early-stage, flat, tail melanoma just post-punch biopsy at the tumour margin; punch biopsy tissue with green Ras^G12V^eGFP tissue adjacent to healthy tissue is shown in (K’).
L–L’’ Multichannel view of the repairing wound illustrating how, 24 h post-wounding, neutrophils (red) have been drawn to both tumour and healthy tissue wound edge. (L) and (L’’) are magnifications of the wound edges of non-tumour and tumour tissue, respectively.
M–M’’ Multichannel view of the repairing wound at 3 days post-wounding, demonstrating that neutrophils have largely resolved away from the wound site in healthy tissue (M’) but remain highly concentrated in wounded tumour tissue (M’’). Data information: Scale bars represent 2 mm (A, B, F-F’’ and K), 50 μm (H, J and J’) and 200 μm (G, I, K’, L-L’’ and M-M’’).

In our previous studies, we observed that zebrafish expressing a mutant Ras^G12V^ oncogene in their skin tend to develop invasive melanomas at sites that are prone to friction and other damage, for example on the ventral fin and tail fin (Feng *et al*, 2010) (Supplementary Fig S1D and E). We wanted to test whether this correlation reflected a true causal association, and so we repeatedly wounded the tail fin of one group of juvenile Ras^G12V^ fish fortnightly for 3 months and compared tumour outcome with equivalent siblings that had not been wounded. By the second round of fortnightly wounds, we saw a clear increase in pigment intensity in the wounded tail fins (Fig1A and B), reflecting recruitment and/or local proliferation of pre-neoplastic melanocytes. This increase in pigmentation, as measured by threshold analysis (Fig1C and D), appeared to reach a plateau after five rounds of wounding, and we left the fish unwounded after six wounds. By 18 months after the initial wounding, we observed that 43% of all wounded fish had developed melanoma at the site of wounding while none of the unwounded fish exhibited fin tip melanomas (Fig1A, B and E), suggesting that chronic wounding can indeed increase the likelihood of cancer growth in tissues that already have a predisposition to developing melanoma. This chronic wounding study was repeated in adult 3- to 6-month-old fish and showed the same result (Supplementary Fig S2A–D).

### Cancer surgery triggers a wound inflammatory response and subsequent influx of neutrophils and macrophages to regions of remaining cancer

Since cancer surgery is an instance whereby a wound is inflicted in the vicinity of a growing cancer, we wondered how a single surgery might impact on these cancer cells. In particular, we wanted to examine how the acute wound inflammatory response which is triggered at any site of tissue injury might draw immune cells to the cancer cells and what the consequences of this might be. The time course of recruitment of inflammatory cells to a tail wound in control fish, without cancer, begins with a first influx of neutrophils (LysC^+^ cells) within an hour of wounding, with numbers peaking at 24 h post-injury; while resident macrophage (mpeg^+^ cells) numbers only begin to increase as neutrophil numbers are diminishing at 6 or 7 days post-wounding, precisely as previously reported for adult fin regeneration studies (Petrie *et al*, 2014) (Supplementary Fig S3H). To investigate adult cancer surgery, we used two models. In one of these, we cut a segment from large melanomas on the tail fin of adult fish and subsequently examined the *in situ* remaining portion of tumour 3 days later (Fig1F–J). Immunostaining of the initially removed cancer reveals the presence of low levels of neutrophils (Fig1G), and staining for phospho-histone H3 shows an associated low level of cell proliferation (Fig1H). At 3 days post-surgery, the remaining region of cancer appears heavily populated with neutrophils (Fig1I), and sections of this region show an associated increase in phospho-histone H3 staining (Fig1J), suggesting that local tissue proliferation might be triggered at any site of surgery because of the associated inflammatory influx. To more clearly image neutrophil influx post-wounding in adult tissues, we selected smaller, flatter melanomas and made punch biopsies in these to include both tumour and healthy tissue (Fig1K). Whole-mount imaging of the initially removed biopsy reveals some neutrophils throughout the melanoma right up to the interface between cancer and healthy tissue (Fig1K’), which reflects previously documented histopathological observations of surgically removed human cancers (Galdiero *et al*, 2013). At 24 h post-biopsy, we see significant recruitment of neutrophils to both healthy and tumour wound edge (Fig1L); however, after 3 days, neutrophils have resolved away from the healthy wound edge but remain at high levels throughout the wounded tumour tissue (Fig1M).

### Live imaging in translucent larvae reveals neutrophils resolving from wounds and recruited to adjacent pre-neoplastic cells

**Figure 2 fig02:**
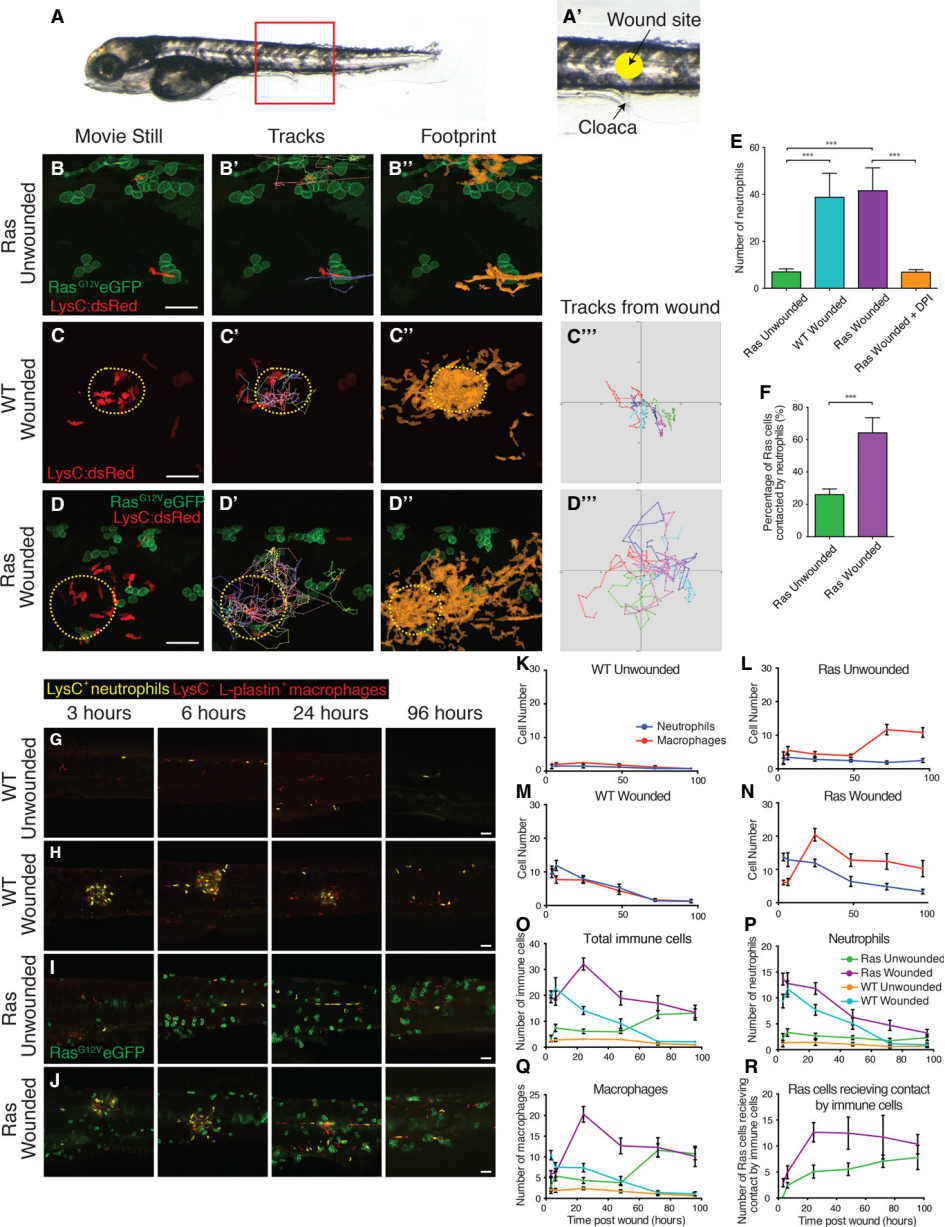
Live imaging reveals neutrophils distracted away from wounds due to competing signals from adjacent pre-neoplastic cells A, A’ Five-days post-fertilisation larva illustrating the region of flank where we image and wound (A). Wounds (yellow circle) are made in the centre of the flank just above the cloaca (arrow) in all larval experiments (A’).
B–B’’ Stills from a time-lapse movie of a larva with Ras^G12V^eGFP pre-neoplastic cell clones but no wound.
C–C’’’ Equivalent time-lapse stills of a control, laser-wounded larva with no pre-neoplastic cell clones at 90 min post-wounding (wound indicated with yellow dotted line).
D–D’’’ Stills from a time-lapse movie of a larva with Ras^G12V^eGFP pre-neoplastic cell clones (green), again wounded 90 min before.
E Graph comparing the number of LysC:dsRed^+^ neutrophils recruited over a 2-h period to the equivalent flank region of unwounded Ras^G12V^eGFP larvae (*n* = 11), versus wounded WT larvae (*n* = 3), wounded Ras^G12V^eGFP larvae (*n* = 5) and wounded Ras^G12V^eGFP larvae treated with DPI inhibitor (*n* = 7). ***P ≤ 0.001.
F Graph comparing the percentage of Ras^+^ pre-neoplastic cells that receive contacts with neutrophils during the 2-h period of the movie in unwounded (*n* = 11) versus wounded (*n* = 5) larvae. ***P ≤ 0.001.
G–J Unwounded WT sibling; LysC:dsRed^+^ larva (*n* = 10 per time point) for comparison of clone growth with (H) laser-wounded WT sibling: LysC:dsRed^+^ larva (*n* = 15 per time point), (I) unwounded Ras^+^; LysC:dsRed^+^ larvae (*n* = 15 per time point) and (J) laser-wounded Ras^+^; LysC:dsRed^+^ (*n* = 20 per time point). Larvae were harvested and fixed between 3 dpf and 7 dpf (i.e. between 3 and 96 h post-wounding), and stained with anti-L-plastin and anti-RFP antibodies to distinguish neutrophils (yellow) and macrophages (red).
K–N Graphs showing the numbers of neutrophils and macrophages in the flanks of unwounded WT siblings (K), unwounded Ras^+^ larvae (L), wounded WT siblings (M) and wounded Ras^+^ larvae (N).
O–Q Graphs indicating the total number of innate immune cells (O), macrophages (P) and neutrophils (Q) recruited over time in wounded and unwounded WT and unwounded and wounded Ras^+^ larvae.
R Graph showing the number of pre-neoplastic cells receiving contact by immune cells in unwounded/wounded larvae over time (*P* = 0.0052 for 5 days post-wound). Data information: In (K–R) *x*-axes denote time post-wound (h). All scale bars represent 50 μm. (B, C and D) show stills of the time-lapse movie; (C’ and D’) show tracks of five immune cells as they migrate after entering the wound; (B’’, C’’ and D’’) show the footprints of all LysC:dsRed immune cells throughout the movie. (C’’’ and D’’’) illustrate the migration of neutrophils from when they enter the centre of the wound. All graphs display mean ± SEM.

To gain a more dynamic impression of how wounding may influence the behaviour of innate immune cells in the vicinity of cancer cells, we made a series of laser wounds adjacent to clones of pre-neoplastic cells on the flanks of zebrafish larvae (Fig2A and A’) which are amenable to live imaging because of their translucency. Our previous studies have shown how fluorescently labelled neutrophils and macrophages are recruited to pre-neoplastic goblet cells expressing mutant Ras^G12V^ and GFP (Feng *et al*, 2010). These innate immune cells are recruited by stochastic pulses of hydrogen peroxide (Feng *et al*, 2010), the same signal that has been shown to draw neutrophils to wounds (Niethammer *et al*, 2009), and they remain at one pre-neoplastic clone for brief periods before moving on to visit adjacent clones (Fig2B, Supplementary Movie S1). Tissue wounding triggers an acute, rapid recruitment of large numbers of neutrophils to the wound, but rather than remaining predominately within the wound site for up to 3 or 4 h, as in control wounded fish with no burden of pre-neoplastic cells (Fig2C, Supplementary Movie S2), many of these immune cells are distracted from the wound and “visit” the nearby pre-neoplastic cells (Fig2D, Supplementary Movie S3); this is most clearly visualised by “footprints” of neutrophil tracks from 90 min to three hours post-wounding which extend well beyond the wound site in fish carrying a pre-neoplastic cell burden, whereas they remain in the vicinity of the wound in fish without pre-neoplastic cells (Fig2C’’ and 02D’’). If wound-triggered hydrogen peroxide release is blocked by treatment with DPI, then few, if any, neutrophils are drawn to the wound (Fig2E), and consequently, many fewer visits to nearby pre-neoplastic cells are seen (Supplementary Movie S4). Because wounding standardly draws many more neutrophils to the flank than are normally present (Fig2E), this leads to considerably more opportunity for contacts with pre-neoplastic cells. Indeed, in this period following wounding, we observe 64.2% of Ras^+^ cells adjacent to a wound receive neutrophil contacts compared to only 26% of pre-neoplastic cells in a comparable region of an unwounded larvae (Fig2F). Contacts ranged from less than one minute to more than 90 min, and we have used them as a proxy for neutrophil/pre-neoplastic cell interactions although we have no evidence that physical contacts between these two lineages are necessary for one cell to influence the other. To follow neutrophil and macrophage recruitment over a substantially longer period, we fixed Ras^+^ larvae and their WT siblings at various time points up to 5 days post-wounding (Fig2G–J). To distinguish macrophages, we immunostained larvae with L-plastin, which is known to be a pan-leucocyte marker in zebrafish larvae, and considered cells which were L-plastin^+^ but LysC^−^ to be macrophages (Feng *et al*, 2010; Jones *et al*, 2013). Over the 5 days post-wounding, the number of neutrophils recruited to flank pre-neoplastic cells in wounded Ras^+^ larvae was significantly higher (*P* = 0.007) than in unwounded Ras^+^ larvae (Fig2P). In contrast, despite the large numbers of macrophages recruited to the wound by 24 h post-wounding, there appears to be no significant increase in macrophage recruitment to nearby pre-neoplastic cells when comparing Ras^+^ unwounded and Ras^+^ wounded larvae over the total time course (Fig2Q; *P* = 0.1019). However, we do observe increased recruitment of macrophages to wounds in Ras^+^ larvae compared to their WT siblings (Fig2Q; *P* = 0.0493), and once recruited, they appear to persist in the area around the wound for longer than in WT siblings due to the presence of the pre-neoplastic cells (Fig2M and N). From the earliest time points examined in fixed larvae—3 h after wounding—we observe a significant increase in the number of pre-neoplastic cells receiving contacts from immune cells (*P* = 0.0052 for 5 days post-wound); the number of these contacts peaks by 24 h post-injury and is maintained for at least 3 days post-wounding (Fig2R).

### Wounding and the associated increase in inflammatory response appear to trigger increased proliferation by pre-neoplastic cells

**Figure 3:**
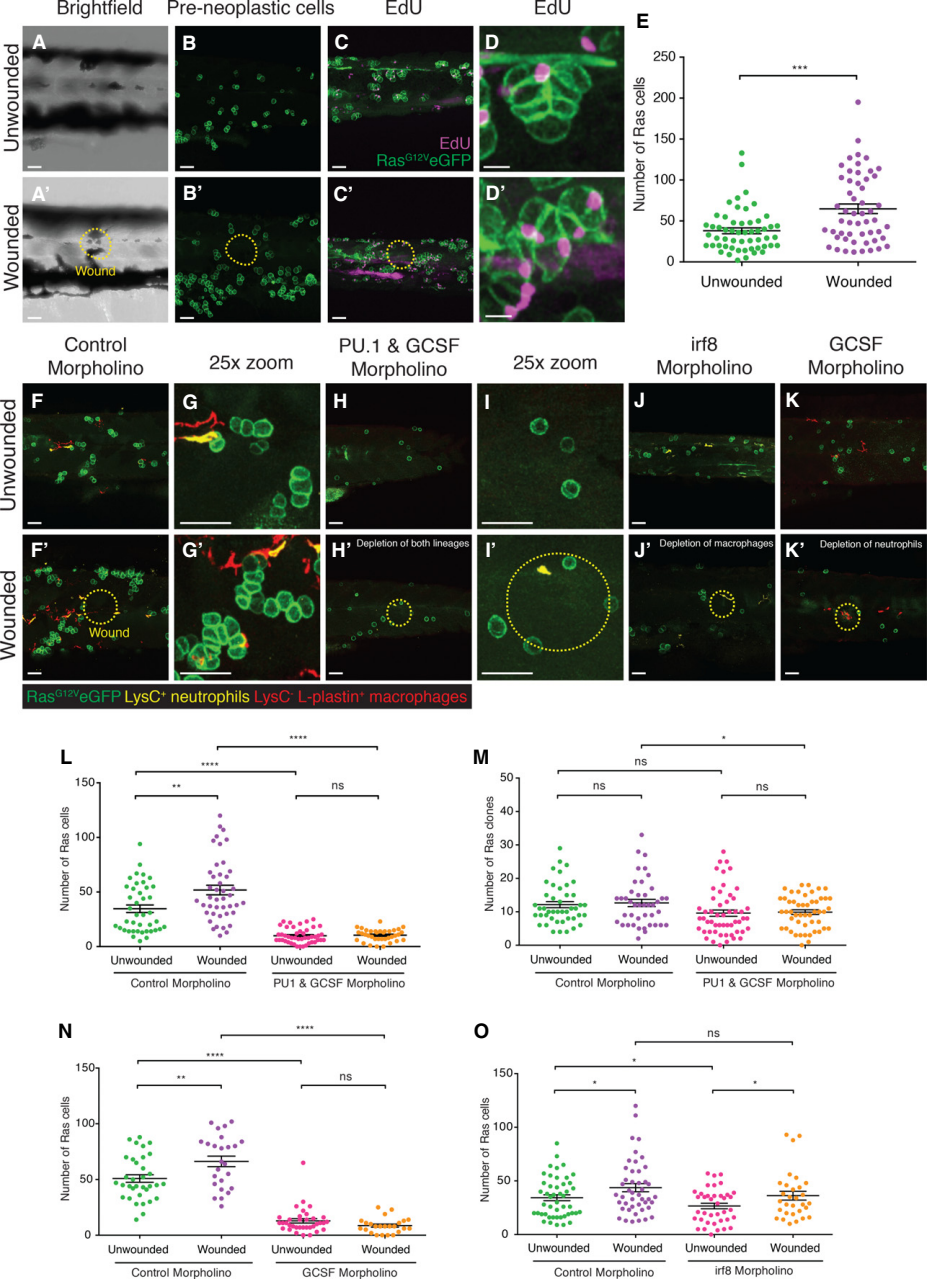
Wounding leads to increased proliferation of pre-neoplastic cells A–D’ Ras^G12V^eGFP larvae were left unwounded (A–D) or laser-wounded (yellow dotted line) at 2 dpf just dorsal to the cloaca (A’–D’). Larvae were left to grow for 3 days before being fixed and analysed for pre-neoplastic cell number (*n* = 30 larvae in each group). EdU accumulation (purple) is shown at low magnification (C and C’) and a representative pre-neoplastic cell clone is shown (D and D’).
E Graph illustrating pre-neoplastic cell numbers in unwounded versus wounded larvae (*n* = 27 of each group). Data from three independent experiments showed the same level of significance. ***P ≤ 0.001.
F–G’ Images of larvae injected with control morpholino at the one-cell stage, and either left unwounded (F, high magnification: G) or laser-wounded at 3 dpf (F’, high magnification: G’) and fixed at 5 dpf.
H–J’ Larvae injected with a combination of PU-1 and GCSF morpholinos at the one-cell stage, left unwounded (H, high magnification: I) or laser-wounded at 3 dpf (H’, high magnification: I’) and fixed at 5 dpf. Images of larvae injected with irf8 morpholino, and either unwounded (J) or laser-wounded at 3 dpf (J’) before subsequent fixation at 5 dpf.
K, K’ Larvae injected with GCSF morpholino, and either left unwounded (K) or laser-wounded at 3 dpf (K’) and fixed at 5 dpf.
L, M Graphs to show the total number of Ras^+^ cells and clones (respectively) at 5 dpf after PU-1 and GCSF morpholinos.
N, O Graphs showing the number of Ras^+^ cells at 5 dpf after irf8 morpholino (N) or after GCSF morpholino injection (O). Data information: All scale bars represent 50 μm except (D) and (D’) which represent 15 μm. *n* = 15–20 larvae in each group (L–O). All graphs display mean ± SEM.

To determine what effect this increased inflammation might have on pre-neoplastic cell proliferation, we counted the number of pre-neoplastic cells 3 days post-wounding in a series of domains extending up to 250 μm away from the wound centre and corresponding approximately to the region of increased inflammation. We see a significant increase in pre-neoplastic cell number in comparison with equivalent flank regions of unwounded larvae (Fig3A, B and E) in bands extending 50–150 μm and 150–250 μm from the wound centre (Supplementary Fig S4), and this is corroborated by a significant increase in EdU-positive pre-neoplastic cells in 2-days post-wounding larvae (Fig3C and D, Supplementary Fig S5A), where the thymidine analogue EdU has been incorporated into the DNA of proliferating cells. This effect is local, rather than systemic, because we see no significant increase in numbers of Ras^+^ cells in the epithelium beyond 250 μm from the wound centre (Supplementary Fig S4), or overlying the yolk or in the head (Supplementary Fig S5). Moreover, only wounds that are sufficiently large to trigger a wound inflammatory response over 48 h appear to trigger a pre-neoplastic proliferative response; small wounds show no increase above background, unwounded, levels (Supplementary Fig S5C). To further test whether this increase is a consequence of the wound inflammatory response, we transiently delayed innate immune cell development by injection of PU.1 and GCSF morpholinos at the one-cell-stage embryo. This led to an almost complete depletion of neutrophils and macrophages until 4 days post-fertilisation (dpf) (Feng *et al*, 2012) and, as a consequence, we observed a significant decrease in pre-neoplastic cell numbers, suggesting reduced proliferation both in unwounded control fish, as previously reported (Feng *et al*, 2010), and in 2-days post-wounded fish, by comparison with fish with a normal wound inflammatory response (Fig3L).

### Wound-associated inflammation drives clonal growth rather than initiation of new clones

The larger number of pre-neoplastic cells in the vicinity of a wound could be due to increased initiation of new clones or increased cell division within clones, or contributions from both of these. However, it is clear that the numbers of clones counted at 2 days after wounding are generally unaltered by comparison with unwounded fish; rather, the increase in neoplastic cell number is almost entirely due to increase in the size of individual clones (Fig3M). Time-lapse studies of individual fish over a period of several days confirm this and indicate that the biggest relative change in clonal size at the wound occurs at about 2 days post-wounding (Supplementary Fig S6). Such studies also reveal some movement of clones towards the wound during the repair process, likely a passive behaviour reflecting that of their healthy epithelial neighbours as the wound closes.

Depleting innate immune cells by morpholino knockdown of PU.1 and GCSF leads to similar numbers of clones—usually around 10 clones within the wound region—but many of these remain as single cells even after wounding. This strongly suggests that innate immune cells are indeed delivering trophic signals to clones of pre-neoplastic cells and encouraging proliferation, rather than delivering factors that initiate new clones.

### Wound-associated neutrophils are responsible for driving increased proliferation

We were able to further dissect the role of neutrophils versus macrophages in this wound inflammatory-driven pre-neoplastic cell proliferation using morpholinos against GCSF, to delay the development of neutrophils (Liongue *et al*, 2009) (Fig3J), and irf8 which blocks the development of macrophages (Li *et al*, 2011) (Fig3K). Delaying the development of neutrophils significantly depleted pre-neoplastic cell numbers, and there was no observed increase in pre-neoplastic cell number after wounding (Fig3N). However, when macrophages were depleted (with a compensatory increase in neutrophils), the numbers of pre-neoplastic cells were only partially reduced, and there was an observed increase after wounding (Fig3O).

### PGE_2_ is part of the signal that drives wound-inflammation-mediated pre-neoplastic cell proliferation

**Figure 4:**
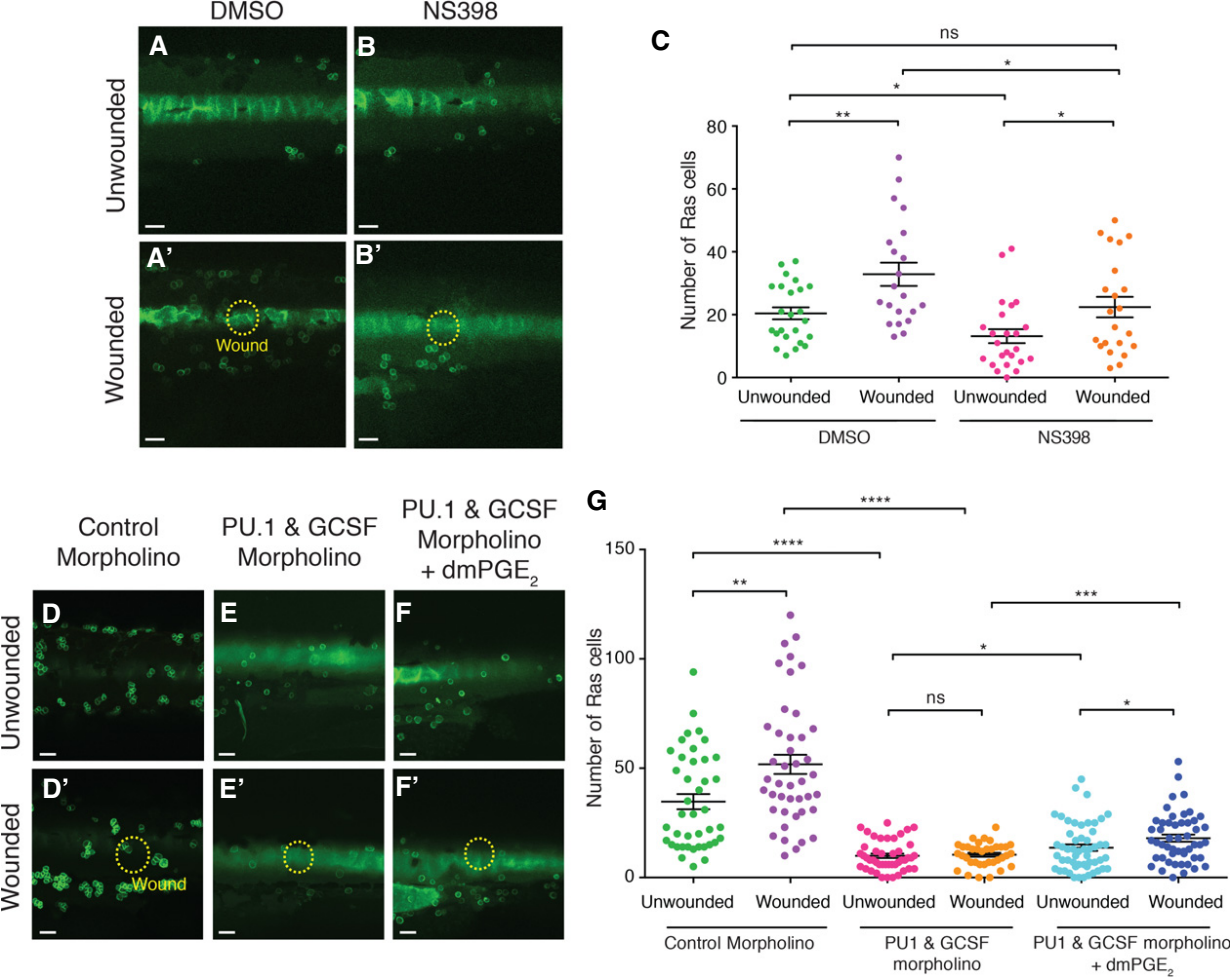
PGE_2_ is one component of the trophic signal driving wound-inflammation-triggered pre-neoplastic cell proliferation A–B’ Images of Ras^+^ larval flanks from control fish bathed in Danieau’s solution or with 10 μM NS398 to block Cox-2 enzyme activity, and left unwounded (A, B) or laser-wounded (A’, B’) at 3 dpf before subsequent fixation at 5 dpf.
C Graph showing quantification of pre-neoplastic cell numbers at 5 dpf from (A–B’) (*n* = 15–20 larvae in each group).
D, D’ Unwounded control Ras^+^ larvae (D) versus sibling larvae that have been laser-wounded at 2 dpf (D’).
E–F’ Unwounded versus wounded larvae after injection with PU-1 and GCSF morpholinos (E and E’), and addition of 20 μM dmPGE_2_ (F and F’).
G Graph showing pre-neoplastic cell numbers from (D–F’). Data information: All scale bars represent 50 μm. Graphs display mean ± SEM.

Since we have previously shown that PGE_2_ is released by neutrophils and macrophages when recruited to pre-neoplastic cells in unwounded larvae (Feng *et al*, 2012), we wondered whether this factor might also be, in part, responsible for the wound-inflammation-triggered local proliferation of these cells. To test this, larvae were immersed in either 0.5% DMSO or 10 μM Cox-2 inhibitor NS398 with 0.5% DMSO and the number of pre-neoplastic cells was analysed 2 days post-laser wounding (Fig4A–C). Although the addition of NS398 does not completely block the increase in pre-neoplastic cell number post-wounding, we now see a significant reduction in numbers, such that there is no significant difference between the unwounded DMSO-treated larvae and the wounded NS398-treated larvae, suggesting that the addition of a Cox-2 inhibitor could partially negate the trophic impact of a cancer surgery (Fig4C). 20 μM of synthetic prostaglandin E_2_ (dmPGE_2_) was added at the point of wounding and largely rescued the proliferative response in immune-depleted zebrafish larvae (Figs3L and 4G), suggesting that PGE_2_ derived from immune cells is one contributor towards the trophic factors responsible for the wound-induced increase in proliferation.

### Infiltration of neutrophils correlates both with extent of ulceration and with tumour cell proliferation in human melanoma

**Figure 5:**
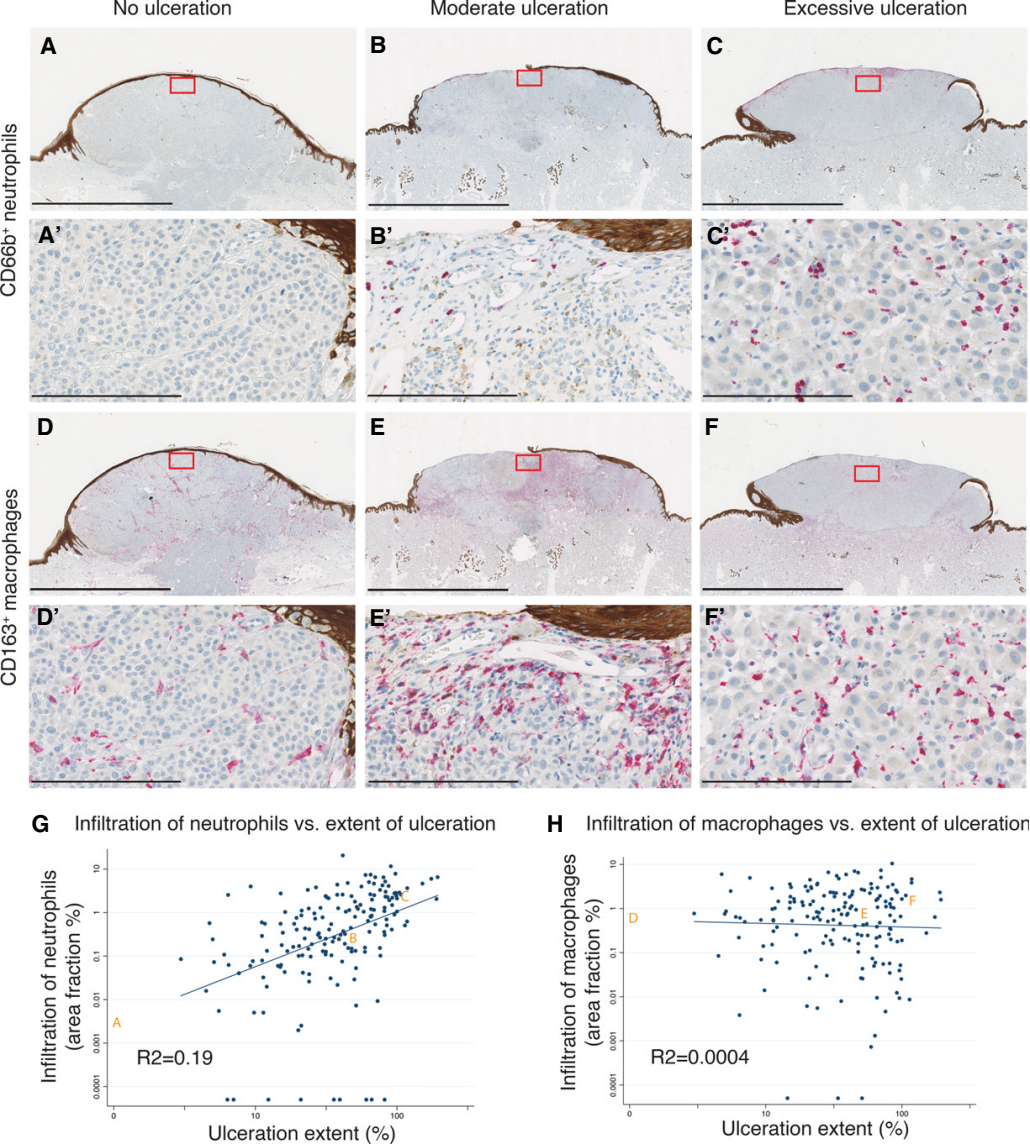
Neutrophil, but not macrophage, recruitment is correlated to the presence and to the extent of ulceration in human melanoma A–C’ Typical non-ulcerated, moderate and excessively ulcerated melanomas, respectively, all immunostained for pan-cytokeratin (brown) to illustrate epidermal wound margins. (A’, B’ and C’) High magnification details from (A, B and C) (highlighted by red boxes) co-stained for CD66b to illustrate neutrophil accumulation.
D–F’ Parallel sections from the same patient blocks as in (A-C’), but stained for CD163 and pan-cytokeratin to illustrate macrophage influx.
G Graph showing the correlation between extent of ulceration and extent of neutrophil influx (*P* <  0.0001; *R*^2^ = 0.19). A, B and C indicate the patient points from whom corresponding neutrophil immunostaining data are shown.
H In contrast to (G), no correlation between extent of ulceration and extent of macrophage influx was observed (*P* = 0.9; *R*^2^ = 0.0004). D, E and F indicate the patient points from whom corresponding macrophage immunostaining data are shown. Data information: Scale bars represent 3 mm (A and D), 6 mm (B, C, E and F) and 200 μm (A’, B’, C’, D’, E’ and F’).

**Figure 6:**
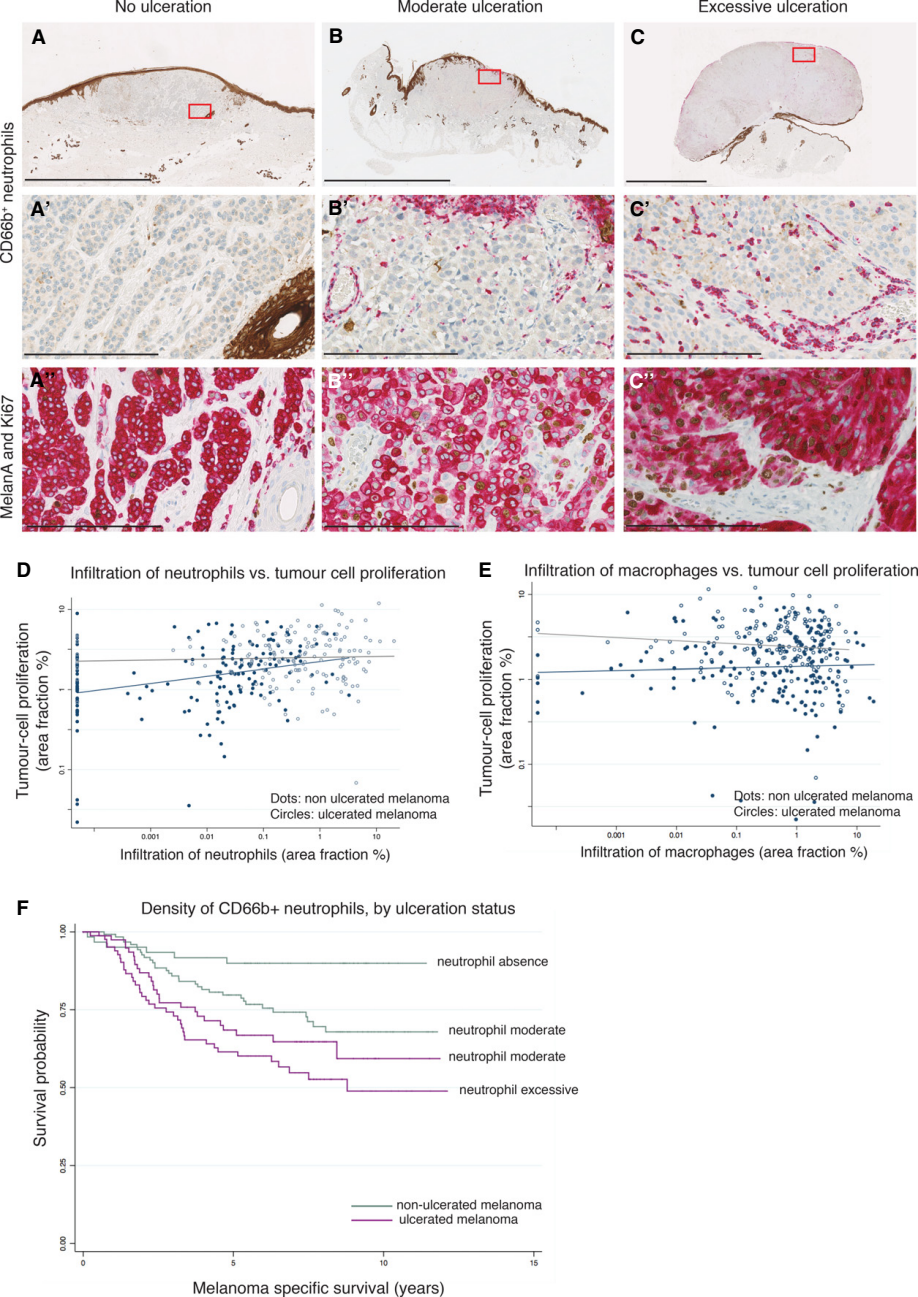
Ulcerated human melanomas show a correlation between level of neutrophil influx, tumour cell proliferation and prognostic outcome A–C’’ Typical non-ulcerated, moderate and excessively ulcerated melanomas, respectively, all immunostained for pan-cytokeratin to illustrate epidermal wound margins. (A’, B’ and C’) High magnification details from (A, B and C) (highlighted by red boxes) co-stained for CD66b to illustrate neutrophil accumulation. (A’’, B’’ and C’’) Parallel sections from the same patient blocks, but stained for MelanA and Ki67 to determine proliferating melanoma cells.
D Graph illustrating the extent of correlation between neutrophil influx and tumour cell proliferation (*P* = 0.002; *R*^2^ = 0.17). Solid dots are from non-ulcerated lesions and show a good correlation, whereas circles represent ulcerated melanomas.
E Graph showing considerably less correlation between macrophage influx and tumour cell proliferation (*P* = 0.56; *R*^2^ = 0.10).
F A series of Kaplan–Meier curves illustrating the link between neutrophil influx (total absence, moderate infiltration (<75 percentile)) and excessive infiltration (>75 percentile) and prognostic outcome in patients with non-ulcerated and ulcerated melanomas, respectively. Data information: Scale bars represent 3 mm (A), 6 mm (B), 10 mm (C) and 200 μm (all other panels).

While there is considerable anecdotal evidence for wound-exacerbated cancer progression (Hofer *et al*, 1998), our zebrafish studies prompted us to investigate whether this association was linked to innate immune cell influx at the wound site. It is already established that ulceration of melanoma is a bad prognostic indicator (Balch *et al*, 2011), and so we examined whether this might be due to wound inflammation. Haematoxylin and eosin staining of sections allows us to delineate the extent of the epidermal wound and to categorise melanomas as having no ulceration, minimal/moderate ulceration (<70% of total tumour length) or excessive ulceration (>70% of tumour length) (In ‘t Hout *et al*, 2012) (Fig5). Our co-staining for CD66b^+^ neutrophils and CD163^+^ macrophages and automated software for quantification of leucocyte numbers reveal a 15 times increase in neutrophils from non-ulcerated to minimal/moderate ulcerated lesions and 100 times increase from non-ulcerated to excessively ulcerated lesions (Fig5A–C and G), whereas there is no such correlation with macrophage numbers and ulceration (Fig5D–F and H, Supplementary Table S1). We observe that recruited neutrophils can be located throughout the melanoma, but are often associated with the superficial wound site in ulcerated lesions (Fig5B and C). We see also a significant correlation between tumour cell proliferation [as assessed by Ki67/MelanA double staining (Nielsen *et al*, 2013)] and extent of neutrophil influx (Fig6A’, B’, C’ and D). This association appears strongest only up to minimal/moderate ulcers, suggesting that melanoma may reach a proliferation plateau soon after initial ulceration when they first become infiltrated by neutrophils, although we have no means to determine at which time point this might have occurred relative to our biopsies. We see no significant correlation between macrophage numbers and tumour cell proliferation (Fig6E and Supplementary Table S2).

### Infiltration of neutrophils further refines the prognostic indication for melanoma in ulcerated lesions

The thicknesses of the primary melanoma, the presence of ulceration and sentinel node status are all well-established prognostic factors in melanoma (Balch *et al*, 2009, 2011). These factors each have independent prognostic impact in our study cohort (Bønnelykke-Behrndtz *et al*, 2014) and have been adjusted for in our multivariate analysis. However, in the current study, we find that the density of CD66b^+^ neutrophil infiltration is an additional, independent prognostic marker of poor melanoma-specific survival. We see a significant interaction with the extent of ulceration, with neutrophil density associating with a poorer prognosis in non-ulcerated and excessively ulcerated tumours compared with minimal/moderate ulcerated tumours (Supplementary Table S3). Interestingly, Kaplan–Meier survival analysis showed that an increase in the fraction of infiltrating neutrophils further refines the prognostic impact of ulceration and separates both the non-ulcerated and ulcerated group of patients (Fig6F). Previous studies have found ambiguous association between macrophage influx and melanoma prognosis, and our study too suggests no significant link between CD163^+^ macrophage numbers and prognostic outcome (Supplementary Table S3).

## Discussion

To study the impact of wounding and, in particular, of the wound inflammatory response, upon cancer initiation and progression, we have turned to a genetically tractable and translucent model, the zebrafish, to live image interactions between innate immune cells and pre-neoplastic cells at sites of tissue damage. We show that continual, chronic wounding of tissues with a genetic predisposition to melanoma leads to an increase in tumour incidence and that even a single acute wound can lead to significantly increased immune cell/pre-neoplastic cell interactions in the local environment of the wound. These interactions trigger an increase in proliferative index in pre-neoplastic cells in the proximity of the wound due to increased inflammation, and we show that this trophic effect is, in part, dependent on PGE_2_ because enhanced growth of pre-neoplastic cells can be partially restored after immune cell depletion by administering PGE_2_ to the medium. In line with the results from our zebrafish experiments, we observe that neutrophil influx associates with increased tumour cell proliferation in human melanoma and also serves as a negative prognostic marker for patient survival.

### Chronic versus acute, and big versus small wounds

That chronic tissue damage and its associated chronic inflammatory response might be fundamental contributors to cancer progression is now well established (Arwert *et al*, 2010) and, indeed, inflammation is now considered one of the ten hallmarks of cancer. Many pathologies of organs involving chronic inflammation are associated with malignancy (Hanahan & Weinberg, 2011), such as the progression of chronic skin wounds to squamous cell carcinoma, otherwise known as Marjolin’s ulcer (Kerr-Valentic *et al*, 2009). Less clear is how acute, rather than chronic, inflammatory episodes might impact on cancer. Our studies indicate that if an acute wound causes sufficient tissue damage to trigger a significant inflammatory response, then those recruited immune cells will likely be drawn to adjacent pre- or later stage neoplastic cells and influence their subsequent fate. In the pre-neoplastic cells that we investigate in zebrafish larvae, this led to an increased proliferative index with a clear increase in clone size, rather than an increase in number of clones, and this is reflected in our clinical studies of ulcerated melanoma where we see an association between extent of wound inflammation and cancer cell proliferation.

The size of the wound is clearly important; this has been previously shown in Rous sarcoma-infected chickens where a large wound, but not a needle wound, was a sufficient trigger for tumour formation (Sieweke *et al*, 1989), and more recently in a mouse model predisposed to develop papillomas where there was a linear correlation between wound size and tumour incidence (Hoste *et al*, 2015). Our studies also show that wounding, per se, does not lead to cancer initiation/progression because when wounds are too small to trigger a significant inflammatory response, or in larger wounds where the immune cells have been deleted, we see no subsequent increase in pre-neoplastic cell proliferation. The size of the ulceration in human melanoma has also been shown to have an important impact on survival, with larger ulcerations associated with poorer melanoma-specific survival (In ‘t Hout *et al*, 2012; Bønnelykke-Behrndtz *et al*, 2014). We hypothesise that at least in part, this might be due to the influx of neutrophils as we show in this study.

### A role for neutrophils in guiding cancer cell growth

To date, macrophages have generally been considered the lead players of all innate immune cells during the later stages of cancer progression and onwards to metastasis (Condeelis & Pollard, 2006). While not excluding a role for macrophages, our zebrafish data and associated clinical melanoma studies suggest that, where there is a tissue damage-associated inflammatory cell influence on adjacent cancer cells, the key innate immune cell might be the neutrophil; only when we delete this lineage, do we see a reduction in pre-neoplastic cell proliferation around the wound, whereas reducing macrophages exerts no significant reduction in pre-neoplastic cell growth, although our macrophage depletion experiments did lead to increased numbers of neutrophils which could be compensatory. These observations of neutrophil influx post-tissue damage are reflected in our clinical studies of “wounded” melanomas, where we see no significant association between degree of macrophage infiltration and proliferative index, whereas neutrophil numbers significantly correlate with increased proliferative index at the melanoma ulcer site and with long-term prognostic outcome. Moreover, there is evidence in other cancers that neutrophil influx may reflect a marker for poor outcome (Donskov, 2013).

Neutrophils are known to infiltrate tumours and can form a significant component of the inflammatory tumour microenvironment (Sionov *et al*, 2014). Tumour-associated neutrophils (TANs) have been shown to promote angiotropism (Bald *et al*, 2014) and to suppress the anti-tumour immune response from T lymphocytes. Indeed, depletion of TANs can lead to tumour regression (Pekarek *et al*, 1995). A recent study shows a direct association between neutrophil presence and melanoma metastasis (Bald *et al*, 2014). In contrast to N1-type circulating neutrophils, which are known for their capacity to defend skin wounds from foreign pathogens, TANs are thought to exist in an alternatively activated N2 state. When compared to unstimulated neutrophils or myeloid-derived suppressor cells (MDSCs), TANs show increased expression of chemokines and cytokines (Fridlender *et al*, 2012) and are likely to be producing many more pro-tumour factors than simply PGE_2_ that we have investigated here.

The TAN N2 phenotype is known to be influenced by the TGFβ pathway (Fridlender *et al*, 2009), which is released at sites of skin damage by platelets and keratinocytes and other cells (Yang *et al*, 1999), which might, in part, explain the neutrophil response we observe following wounding. Moreover, N2 neutrophils can be switched back to a tumour-cytotoxic N1 polarity through inhibition of TGFβ (Fridlender *et al*, 2009), or following immunologic or cytokine activation (Kim *et al*, 2008), re-enabling their potential to limit tumour growth (Kim *et al*, 2008), which is likely to be of significant therapeutic relevance. Clearly, it will be important to investigate further both macrophage and neutrophil phenotypic switches and how these are influenced by their exposure to wounds and then to pre-neoplastic cells, although currently this is difficult to analyse in zebrafish due to the lack of M1/M2 and N1/N2 markers.

### Implications for the clinic

Our studies in zebrafish confirm an indication from many anecdotal clinical observations that cancer surgery and biopsy may influence patient outcomes in ways beyond simple removal of cancer tissue (Naumov *et al*, 2009). Although we must be cautious in extrapolating observations in zebrafish larvae to cancer surgery in human patients, it is clear that particularly for surgery that may not remove all the cancer, there must be concerns how the wound inflammatory response might influence remaining cancer cells.

Surgery is still the treatment of choice in localised melanoma, while immunotherapy in the form of the anti CTLA-4 antibody ipilimumab (Hodi *et al*, 2010) has emerged as a standard treatment in the metastatic setting and there are exciting new treatment options with the PD-1 antibodies nivolumab and pembrolizumab in development (Topalian *et al*, 2012; Wolchok *et al*, 2013; Robert *et al*, 2014). A primary melanoma is generally excised in a 2-step procedure with margins, up to 2 cm, depending on the initial thickness. Sentinel lymph node biopsy, for staging and evaluation of micro-metastasis in the regional lymph node basin, is recommended for patients with melanoma thickness over 1 mm or with the presence of ulceration or signs of excessive proliferation (Jensen *et al*, 2012). The value of sentinel lymph node biopsy and subsequent removal of regional lymph nodes when positive for micro-metastasis is questionable, however, as neither confers a survival benefit (Morton *et al*, 2014). In addition, a high false-negative rate, defined as the development of macro-metastasis in the same regional lymph node region after a negative sentinel lymph node biopsy, adds complexity to the discussion. Pathological, surgical and biological factors have been associated with the detection rate of micro-metastasis in the sentinel node (Riber-Hansen *et al*, 2012). However, wound-associated inflammation may have some impact on non-sentinel remnant cells, offering them a favourable niche within the lymph node region, which might support further tumour growth. Sentinel lymph node biopsy is currently accepted as an important tool for proper staging and selection of patients for additional targeted or immune-modulating therapy.

Ulceration of primary melanomas has long been known to reflect a poor prognosis for the patient, but recently it has also been shown that patients with ulceration and microscopic lymph node metastases respond better to interferon than those without ulceration and with macroscopic lymph node metastases (Eggermont *et al*, 2012). This might suggest that while a chronic inflammatory response initially favours the tumour cell, some further treatments may have the potential to switch the chronic inflammatory response to one that drives an anti-tumoural response.

At a fundamental level, our studies suggest a layered approach for developing therapeutics that might counter the effects of the damage-associated acute inflammatory response. A reductionist approach would be to simply delete, or at least dampen, the wound inflammatory response; alternatively, and more subtle, it would be to inhibit the trophic signals delivered to cancer cells by the inflammatory cells. A third strategy would be to take advantage of the enhanced recruitment of inflammatory cells to nearby cancer cells, and encourage the inflammatory cells to kill rather than nurture the cancer cells. Since the acute wound inflammatory response is transient, persisting only until the resolution phase, there will be a limited window post-wounding when wound-attracted immune cells can deliver trophic signals to local cancer cells. Our studies in zebrafish larvae show that blocking one of these trophic signals at the time of wounding can, in part, inhibit the inflammation-dependent proliferative effect. At these stages and for this pre-neoplastic lineage, we know that PGE_2_ is a component of the trophic signal, and consequently, prostaglandin synthesis inhibitors are effective. Clinically, long-term use of prostaglandin inhibitors, especially aspirin, is associated with reduced risk of developing several types of cancer (Rothwell *et al*, 2012). They may also have an adjuvant role in cancer surgery, although their effects on platelet aggregation must also be taken into consideration.

More importantly, this observation highlights how important it is for us to gain a better understanding of what are the key immune cell-derived trophic signals for various cancers, and these may be different if the innate immune cells have been drawn to the cancer, and primed in some way, after previously being part of an acute inflammatory wound response. Prostaglandins are likely to be one of the players, for some cancer cell types, but certainly not the only one, and more research should focus on determining the trophic signals originating from wounds and wound-recruited immune cells which may impact on tumour growth. We need to know considerably more about precisely how immune cells behave in the presence of pre-neoplastic and cancer cells with various genetic lesions (beyond RasV12 as we have investigated in our zebrafish studies), particularly after they have previously responded to wound cues, before considering how to modulate these behaviours in ways that might benefit the patient.

### Summary

The overall implication of this study is not that surgery or biopsy collection should be avoided, but rather that the cellular processes that occur as a consequence of local wounding, particularly the wound inflammatory response, require improved understanding as surgical wounds may have unintended consequences. In this regard, it is likely that the move towards more minimal surgery will be beneficial to patients by avoiding unnecessary inflammatory stimulation. A dispersed, infiltrative cancer will still require aggressive surgery to ensure macroscopic clearance, whereas minimal surgery for a contained tumour may reduce inflammatory influx and the negative downstream consequences that we describe in this paper.

The translucency of zebrafish larval tissues has offered us a unique opportunity to live image how the wound inflammatory response impacts on adjacent pre-neoplastic or cancer cells and reveals some interesting insights. These studies show definitive evidence that innate immune cells drawn to wounds can rapidly move on to competing attractants released by cancer cells, and this observation, particularly, the key role played by neutrophils, has guided our investigations in human studies where the presence of these cells may serve as “warning markers” for cancer-promoting influences by acute and chronic wound episodes in and around patient tumours. Moreover, a better understanding of these immune cell/cancer cell interactions at sites adjacent to tissue damage will guide potential perioperative therapeutic intervention where cancer cells might remain after surgery.

## Materials and Methods

### Zebrafish lines and maintenance

Adult zebrafish (*Danio rerio*) were maintained and crossed as previously described (Westerfield, 2007). In brief, adult zebrafish were reared at a constant temperature of 28°C, mated and fertilised eggs were collected, bleached, maintained and staged according to standard protocols (Westerfield, 2007). Strains included: Et(kita:GalTA4,UAS:mCherry)*hzm1* (Et30) (Santoriello *et al*, 2010), Tg(5XUAS:eGFP-H-RASV12)io6 (Santoriello *et al*, 2010), Tg(LysC:dsRed)nz (Hall *et al*, 2007) Tg(mpeg1:FRET) (a kind gift from Nikolay Ogryzko and Stephen Renshaw at the University of Sheffield) and *tp53*^M214K^ (Berghmans *et al*, 2005). All experiments were conducted with local ethical approval from the University of Bristol and in accordance with UK Home Office regulations.

### Morpholinos

All the morpholinos were obtained from (GeneTools LLC) and 100-μm drops (1 nl volume) were injected into one-cell-stage embryos. Morpholinos include: *pu.1* 5′-GATATACTGATACTCCATTGGTGGT-3′ (Rhodes *et al*, 2005); *gcsfr* 5′-GAAGCACAAGCGAGACGGATGCCAT-3′ (Liongue *et al*, 2009); and *irf8* 5′-AATGTTTCGCTTACTTTGAAAATGG-3′ (Li *et al*, 2011).

### Whole-mount immunofluorescence

Whole-mount immunostaining was performed as previously described (Feng *et al*, 2010). Primary antibodies used in this study include rabbit polyclonal anti-L-plastin antibody (1:500) (Feng *et al*, 2010), chicken polyclonal anti-L-plastin antibody (1:200) (Feng *et al*, 2010), mouse polyclonal anti-GFP (1:100) (Abcam, ab13970), rabbit polyclonal anti-RFP (1:200) (MBL), rabbit monoclonal anti-phospho-histone H3 (Ser10) (1:200) (Cell Signalling, 3377) and rabbit anti-Cox2 (1:200) (Cayman Chemicals) (Feng *et al*, 2012). AlexaFluor-488 IgG, AlexaFluor-546 IgG or AlexaFluor-647 secondary IgGs (all from Invitrogen and used at 1:500) were used to reveal primary antibody localisation. L-plastin antibody marks all leucocytes at larval stages, and so to determine macrophage numbers in fixed whole-mount preparation, we counted L-plastin^+^; LysC^−^ cells, as previously described (Feng *et al*, 2010; Jones *et al*, 2013). To measure larval proliferation, a Click-iT EdU imaging kit (Invitrogen) was used. In brief, larvae were treated with 400 μM EdU immediately after wounding at 3 dpf and maintained for 2 days post-wounding when they were fixed, the Click-iT reaction was performed and the larvae were immunostained according to the manufacturer’s instructions. Larvae were then mounted in Citifluor (Agar Scientific) and imaged on a Leica SP5-II AOBS confocal laser scanning microscope attached to a Leica DM I6000 inverted epifluorescence microscope.

### Wounding zebrafish embryos

Larvae were first sorted into Ras^+^ and WT sibling groups and anesthetised in Danieau’s solution containing 0.1 mg/ml tricaine (Sigma). Laser wounds were made as previously described (Redd *et al*, 2006) using a UV-nitrogen laser microbeam coupled to a Zeiss Axioplan 2 microscope (Micropoint Laser System, Photonic Instruments). Typical laser wounds required a 3-s pulse from the Coumarin 440-nm dye cell laser focused through a 40× Achroplan water immersion objective. Larvae were kept for the required post-wounding period, when they were fixed and immunostained as described above. The pre-neoplastic cell numbers in larvae were counted manually (always in the same region above the cloaca) with a 20× objective and 1.5× zoom.

### Pharmacological treatment

Larvae were treated with 30 μM NS398 or 20 μM dmPGE_2_ (both from Cayman Chemicals) immediately after laser wounding at 2 or 3 dpf in Danieau’s solution containing 0.5% DMSO. After treatment, larvae were fixed in 4% PFA overnight, immunostained and imaged as described above. For DPI treatment, larvae were incubated in 100 μM DPI (Sigma) in Danieau’s solution containing 1% DMSO for 45 min prior to wounding and throughout the period of imaging.

### Live imaging of zebrafish larvae

Wounded or control larvae were mounted on their sides in 1.5% low-melting agarose (Sigma), in a glass-bottomed dish, filled with Danieau’s solution containing 0.01 mg/ml tricaine. The climate chamber covering the microscope stage was set at 28°C. Images were collected using a Leica SP5-II AOBS confocal laser scanning microscope attached to a Leica DM I6000 inverted epifluorescence microscope with a 63× glycerol lens. Movies were recorded at either 1 or 2 frames/min and were exported from Volocity as QuickTime movies using the Sorenson3 video compressor to play at 6 frames/s.

### Adult zebrafish surgery and live imaging

Adult zebrafish (either Et30:GalTA4*;* UAS:eGFP-H-RASV12 alone or crossed to *tp53*^M214K^, to increase melanoma incidence) were anesthetised in tank system water containing 0.1 mg/ml tricaine (Sigma). Tumours were excised, or the tip of the tail fin was resected, with a microsurgical knife (World Precision Instruments) on a 2%-agarose plate. Punch biopsies were taken with a 1-mm sterile disposable biopsy punch (Kai Medical). Images were taken using a Leica camera (DFC320) attached to a Leica MZFLIII dissecting microscope. Live confocal imaging was performed on anaesthetised, punch-biopsied fish with their tails mounted in 1.5% low-melting agarose (Sigma) using a Leica SP8 AOBS laser scanning confocal attached to a Leica DM6000 upright microscope with a 10× water immersion lens.

### Adult zebrafish immunohistochemistry

Adult zebrafish tissue was fixed in 4% PFA for 2 h at room temperature or overnight at 4°C, washed in PBS and transferred to PBS plus 30% sucrose at least overnight. Tissues were embedded in Tissue-Tek O.C.T. and frozen in isopentane cooled by liquid nitrogen and 14-μm section cut by a Bright OTS cryostat onto Superfrost Plus microscope slides (VWR). Frozen sections were washed in PBS with 0.1% Triton X-100, blocked and incubated overnight with primary antibody (as above) at 4°C. Slides were subsequently washed extensively with PBS with 1% Triton X-100, re-blocked briefly and secondary antibody added for 2 h at room temperature, before washing in PBS with 0.1% Triton X-100 overnight. Slides were mounted in Mowial or ProLong Gold antifade reagent (Invitrogen) and imaged using a Leica SP5-II AOBS confocal laser scanning microscope.

### Post-image acquisition analysis

The number of pre-neoplastic cell clones, immune cells recruited and the number of pre-neoplastic cell contacts were counted manually. Distances from wounds to pre-neoplastic cell clones were measured using the measure function in Volocity software (Perkin Elmer Improvision). All time-lapse movie quantification and tracking analysis were performed using Volocity. Individual LysC:DsRed^+^ cells were identified using automatic ‘‘find objects use intensity’’ and ‘‘colour the object’’ functions in Volocity for all the time points to generate a ‘‘footprint map’’. Adult zebrafish tails were analysed using the threshold function on ImageJ where images were first changed to an 8-bit image, threshold applied to 130 and the number of particles automatically counted. The area fraction was used to determine the pigmentation of the tail fins, or the accumulation of LysC^+^, mpeg^+^ or L-plastin^+^ immune cells. Montages of adult zebrafish were made using the photomerge function in Adobe Photoshop. In some instances, the notochord was cropped from the data set using Imaris software (Bitplane) for improved visualisation of overlying RasGV12eGFP goblet cells.

### Human tissue collection

Danish patients diagnosed with primary cutaneous melanoma from 2001 to 2008 were retrospectively located from pathology files. Melanomas with verified ulceration (*n* = 207) were matched consecutively with a reference group of non-ulcerated melanoma, matched according to Breslow thickness and age. In total, 385 patients with superficial spreading, nodular- and lentigo-malignant melanomas were included. Data on pathological parameters and follow-up information pertaining to the included patients were collected from electronic patient files, pathology files and the Danish Melanoma Group database. Patients with more than one melanoma, missing follow-up information or re-evaluated as metastatic, satellite or benign lesions were excluded to avoid follow-up bias (*n* = 17). For proper evaluation and computer estimation, tumours with no sufficient tumour tissue left, largely pigmented melanoma or MelanA-negative melanomas were excluded. For the neutrophil, macrophage and proliferation analyses, 180, 179 and 164 patients with verified ulcerated melanoma and 201, 203 and 181 patients with non-ulcerated melanoma were included in the analyses, respectively. The Regional Committee for Health Research Ethics in the Region Middle of Denmark approved the study.

### Melanoma immunohistochemistry

Formalin-fixed and paraffin-embedded sections were serial sectioned at 3 μm.

Commercially available antibodies were used for the immunohistochemistry, with a control section for every batch of 20 sections, and evaluated blinded by a senior pathologist (TS). Ultraview fast red was used as detection system, to minimise the influence of the pigmentation of melanophages or melanoma cells. The sections were stained for cd163^+^ macrophages (Ab Serotec, EDHu-1, 1:100, cytoplasm) and cd66b^+^ granulocytes (BD Bioscience, G10F5, 1:200, cytoplasm). For tumour cell proliferation, we used double stains of MelanA (Cell Marque, M2-7C10, 1:50, cytoplasm) to detect melanoma cells and Ki67 (Spring, sp-6, 1:100, nuclei) as a marker of proliferating nuclei.

### Quantification of melanoma inflammation and proliferation

Objective estimates of the area fraction (%) of infiltrating neutrophils (area intratumoural or intravascular cd66b^+^ neutrophils/area region of interest × 100), macrophages (area intra-tumoural or intra-stromal cd163^+^ macrophages/area region of interest × 100) and tumour cell proliferation (area Ki67^+^  MelanA^+^ cells/area Ki67^+^  MelanA^+^ and area of Ki67^−^ MelanA^+^) were acquired by computer-assisted image analysis and software from Visiopharm A/S, Denmark (Carus *et al*, 2013; Nielsen *et al*, 2013).

### Statistical analysis

Zebrafish data were analysed using Prism 6 software (GraphPad), unpaired two-tailed Student’s *t*-test for comparisons between two groups (Prism 6, GraphPad Software) and one-way ANOVA with appropriate post-test adjustment for multiple group comparisons (following D’Agnostino-Pearson omnibus normality tests). Graphs display mean ± SEM unless otherwise indicated. Statistical significance is indicated on graphs using standard conventions, as follows: ns: *P* > 0.05, *P ≤ 0.05, **P ≤ 0.01, ***P ≤ 0.001, ****P ≤ 0.0001. All experiments were repeated three times (non-randomised or blinded) and were performed on at least 15 larval or 3 adult fish. Replicates are indicated in relevant figure legends.

Human patient data were grouped by ulceration status (absence vs. presence), and if present dichotomised by the extent (relative length minimal/moderate <70% vs. excessive >70% (In ‘t Hout *et al*, 2012)). Data were log-transformed and analysed with linear regression, tested for interactions of ulceration status, ulceration extent, Breslow thickness and sentinel node status. In order to allow log transformation and analysis of sections with no infiltrating neutrophils, zero values were replaced with a defined low value of 0.0005. The assumption of normal distribution was assessed using the residuals and found adequate. Statistical analysis of melanoma-specific survival was performed using the Cox proportional hazards using a robust sandwich covariance matrix estimate to account for the case control design. Breslow thickness, sentinel node status and ulceration, which are all main factors defining the stage, all demonstrated independent prognostic impact in our study cohort and were adjusted for in the multivariate analysis.

## Supporting Information

**Supplementary Figure and Movie Legends**

**Review Process File**

## References

[b1] Arwert EN, Lal R, Quist S, Rosewell I, van Rooijen N, Watt FM (2010). Tumor formation initiated by nondividing epidermal cells via an inflammatory infiltrate. Proc Natl Acad Sci USA.

[b2] Balch CM, Gershenwald JE, Soong SJ, Thompson JF, Atkins MB, Byrd DR, Buzaid AC, Cochran AJ, Coit DG, Ding S, Eggermont AM, Flaherty KT, Gimotty PA, Kirkwood JM, McMasters KM, Mihm MC, Morton DL, Ross MI, Sober AJ, Sondak VK (2009). Final version of 2009 AJCC melanoma staging and classification. J Clin Oncol.

[b3] Balch CM, Gershenwald JE, Soong SJ, Thompson JF (2011). Update on the melanoma staging system: the importance of sentinel node staging and primary tumor mitotic rate. J Surg Oncol.

[b4] Bald T, Quast T, Landsberg J, Rogava M, Glodde N, Lopez-Ramos D, Kohlmeyer J, Riesenberg S, van den Boorn-Konijnenberg D, Homig-Holzel C, Reuten R, Schadow B, Weighardt H, Wenzel D, Helfrich I, Schadendorf D, Bloch W, Bianchi ME, Lugassy C, Barnhill RL (2014). Ultraviolet-radiation-induced inflammation promotes angiotropism and metastasis in melanoma. Nature.

[b5] Berghmans S, Murphey RD, Wienholds E, Neuberg D, Kutok JL, Fletcher CD, Morris JP, Liu TX, Schulte-Merker S, Kanki JP, Plasterk R, Zon LI, Look AT (2005). tp53 mutant zebrafish develop malignant peripheral nerve sheath tumors. Proc Natl Acad Sci USA.

[b6] Biswas SK, Gangi L, Paul S, Schioppa T, Saccani A, Sironi M, Bottazzi B, Doni A, Vincenzo B, Pasqualini F, Vago L, Nebuloni M, Sica A, Mantovani A (2006). A distinct and unique transcriptional program expressed by tumor-associated macrophages (defective NF-kappa B and enhanced IRF-3/STAT1 activation). Blood.

[b7] Bogden AE, Moreau JP, Eden PA (1997). Proliferative response of human and animal tumours to surgical wounding of normal tissues: onset, duration and inhibition. Br J Cancer.

[b8] Bønnelykke-Behrndtz M, Schmidt H, Christensen I, Damsgaard T, Møller H, Bastholt L, Nørgaard P, Steiniche T (2014). Prognostic stratification of ulcerated melanoma; not only the extent matters. Am J Clin Pathol.

[b9] Carus A, Ladekarl M, Hager H, Pilegaard H, Nielsen PS, Donskov F (2013). Tumor-associated neutrophils and macrophages in non-small cell lung cancer: no immediate impact on patient outcome. Lung Cancer.

[b10] Ceelen W, Pattyn P, Mareel M (2013). Surgery, wound healing, and metastasis: recent insights and clinical implications. Crit Rev Oncol Hematol.

[b11] Chang HY, Sneddon JB, Alizadeh AA, Sood R, West RB, Montgomery K, Chi J-T, van de Rijn M, Botstein D, Brown PO (2004). Gene expression signature of fibroblast serum response predicts human cancer progression: similarities between tumors and wounds. PLoS Biol.

[b12] Clark-Lewis I, Murray AW (1978). Tumor Promotion and the Induction of Epidermal Ornithine Decarboxylase Activity in Mechanically Stimulated Mouse Skin. Cancer Res.

[b13] Colleypriest BJ, Ward SG, Tosh D (2009). How does inflammation cause Barrett’s metaplasia?. Curr Opin Pharmacol.

[b14] Combemale P, Bousquet M, Kanitakis J, Bernard P (2007). Malignant transformation of leg ulcers: a retrospective study of 85 cases. J Eur Acad Dermatol Venereol.

[b15] Condeelis J, Pollard JW (2006). Macrophages: obligate partners for tumor cell migration, invasion, and metastasis. Cell.

[b16] Donskov F (2013). Immunomonitoring and prognostic relevance of neutrophils in clinical trials. Semin Cancer Biol.

[b17] Dvorak HF (1986). Tumors: wounds that do not heal. N Engl J Med.

[b18] Eggermont AM, Suciu S, Testori A, Kruit WH, Marsden J, Punt CJ, Santinami M, Sales F, Schadendorf D, Patel P, Dummer R, Robert C, Keilholz U, Yver A, Spatz A (2012). Ulceration and stage are predictive of interferon efficacy in melanoma: results of the phase III adjuvant trials EORTC 18952 and EORTC 18991. Eur J Cancer.

[b19] Feng Y, Santoriello C, Mione M, Hurlstone A, Martin P (2010). Live imaging of innate immune cell sensing of transformed cells in zebrafish larvae: parallels between tumor initiation and wound inflammation. PLoS Biol.

[b20] Feng Y, Renshaw S, Martin P (2012). Live imaging of tumor initiation in zebrafish larvae reveals a trophic role for leukocyte-derived PGE(2). Curr Biol.

[b21] Forget P, Vandenhende J, Berliere M, Machiels J-P, Nussbaum BÆ, Legrand C, De Kock M (2010). Do intraoperative analgesics influence breast cancer recurrence after mastectomy? A retrospective analysis. Anesth Analg.

[b22] Fridlender ZG, Sun J, Kim S, Kapoor V, Cheng G, Ling L, Worthen GS, Albelda SM (2009). Polarization of tumor-associated neutrophil phenotype by TGF-beta: “N1” versus “N2” TAN. Cancer Cell.

[b23] Fridlender ZG, Sun J, Mishalian I, Singhal S, Cheng G, Kapoor V, Horng W, Fridlender G, Bayuh R, Worthen GS, Albelda SM (2012). Transcriptomic analysis comparing tumor-associated neutrophils with granulocytic myeloid-derived suppressor cells and normal neutrophils. PLoS ONE.

[b24] Galdiero MR, Bonavita E, Barajon I, Garlanda C, Mantovani A, Jaillon S (2013). Tumor associated macrophages and neutrophils in cancer. Immunobiology.

[b25] Grivennikov SI, Greten FR, Karin M (2010). Immunity, inflammation, and cancer. Cell.

[b26] Hall C, Flores MV, Storm T, Crosier K, Crosier P (2007). The zebrafish lysozyme C promoter drives myeloid-specific expression in transgenic fish. BMC Dev Biol.

[b27] Hanahan D, Weinberg RA (2011). Hallmarks of cancer: the next generation. Cell.

[b28] Hennings H, Boutwell RK (1970). Studies on the mechanism of skin tumor promotion. Cancer Res.

[b29] Hodi FS, O’Day SJ, McDermott DF, Weber RW, Sosman JA, Haanen JB, Gonzalez R, Robert C, Schadendorf D, Hassel JC, Akerley W, van den Eertwegh AJ, Lutzky J, Lorigan P, Vaubel JM, Linette GP, Hogg D, Ottensmeier CH, Lebbe C, Peschel C (2010). Improved survival with ipilimumab in patients with metastatic melanoma. N Engl J Med.

[b30] Hofer SO, Shrayer D, Reichner JS, Hoekstra HJ, Wanebo HJ (1998). Wound-induced tumor progression: a probable role in recurrence after tumor resection. Arch Surg.

[b31] Hoste E, Arwert EN, Lal R, South AP, Salas-Alanis JC, Murrell DF, Donati G, Watt FM (2015). Innate sensing of microbial products promotes wound-induced skin cancer. Nat Commun.

[b32] In ‘t Hout FE, Haydu LE, Murali R, Bonenkamp JJ, Thompson JF, Scolyer RA (2012). Prognostic importance of the extent of ulceration in patients with clinically localized cutaneous melanoma. Ann Surg.

[b33] Jensen TO, Schmidt H, Moller HJ, Hoyer M, Maniecki MB, Sjoegren P, Christensen IJ, Steiniche T (2009). Macrophage markers in serum and tumor have prognostic impact in American Joint Committee on Cancer stage I/II melanoma. J Clin Oncol.

[b34] Jensen TO, Schmidt H, Moller HJ, Donskov F, Hoyer M, Sjoegren P, Christensen IJ, Steiniche T (2012). Intratumoral neutrophils and plasmacytoid dendritic cells indicate poor prognosis and are associated with pSTAT3 expression in AJCC stage I/II melanoma. Cancer.

[b35] Jones RA, Feng Y, Worth AJ, Thrasher AJ, Burns SO, Martin P (2013). Modelling of human Wiskott-Aldrich syndrome protein mutants in zebrafish larvae using in vivo live imaging. J Cell Sci.

[b36] Kasper M, Jaks V, Are A, Bergstrom A, Schwager A, Svard J, Teglund S, Barker N, Toftgard R (2011). Wounding enhances epidermal tumorigenesis by recruiting hair follicle keratinocytes. Sci Signal.

[b37] Kerr-Valentic MA, Samimi K, Rohlen BH, Agarwal JP, Rockwell WB (2009). Marjolin’s ulcer: modern analysis of an ancient problem. Plast Reconstr Surg.

[b38] Kim S, Buchlis G, Fridlender ZG, Sun J, Kapoor V, Cheng G, Haas A, Cheung HK, Zhang X, Corbley M, Kaiser LR, Ling L, Albelda SM (2008). Systemic blockade of transforming growth factor beta signaling augments the efficacy of immunogene therapy. Cancer Res.

[b39] Kuraishy A, Karin M, Grivennikov SI (2011). Tumor promotion via injury- and death-induced inflammation. Immunity.

[b40] Leder A, Kuo A, Cardiff RD, Sinn E, Leder P (1990). v-Ha-ras transgene abrogates the initiation step in mouse skin tumorigenesis: effects of phorbol esters and retinoic acid. Proc Natl Acad Sci USA.

[b41] Lee JW, Shahzad MM, Lin YG, Armaiz-Pena G, Mangala LS, Han HD, Kim HS, Nam EJ, Jennings NB, Halder J, Nick AM, Stone RL, Lu C, Lutgendorf SK, Cole SW, Lokshin AE, Sood AK (2009). Surgical stress promotes tumor growth in ovarian carcinoma. Clin Cancer Res.

[b42] Li L, Jin H, Xu J, Shi Y, Wen Z (2011). Irf8 regulates macrophage versus neutrophil fate during zebrafish primitive myelopoiesis. Blood.

[b43] Lin MV, King LY, Chung RT (2015). Hepatitis C virus-associated cancer. Annu Rev Pathol.

[b44] Liongue C, Hall CJ, O’Connell BA, Crosier P, Ward AC (2009). Zebrafish granulocyte colony-stimulating factor receptor signaling promotes myelopoiesis and myeloid cell migration. Blood.

[b45] Mantovani A, Sica A (2010). Macrophages, innate immunity and cancer: balance, tolerance, and diversity. Curr Opin Immunol.

[b46] Martin P, Shaw TJ (2009). Wound repair at a glance. J Cell Sci.

[b47] Morton DL, Thompson JF, Cochran AJ, Mozzillo N, Nieweg OE, Roses DF, Hoekstra HJ, Karakousis CP, Puleo CA, Coventry BJ, Kashani-Sabet M, Smithers BM, Paul E, Kraybill WG, McKinnon JG, Wang H-J, Elashoff R, Faries MB (2014). Final trial report of sentinel-node biopsy versus nodal observation in melanoma. N Engl J Med.

[b48] Naumov GN, Folkman J, Straume O (2009). Tumor dormancy due to failure of angiogenesis: role of the microenvironment. Clin Exp Metastasis.

[b49] Nielsen PS, Riber-Hansen R, Jensen TO, Schmidt H, Steiniche T (2013). Proliferation indices of phosphohistone H3 and Ki67: strong prognostic markers in a consecutive cohort with stage I/II melanoma. Mod Pathol.

[b50] Niethammer P, Grabher C, Look AT, Mitchison TJ (2009). A tissue-scale gradient of hydrogen peroxide mediates rapid wound detection in zebrafish. Nature.

[b51] O’Byrne KJ, Dalgleish AG (2001). Chronic immune activation and inflammation as the cause of malignancy. Br J Cancer.

[b52] O’Leary D, Wang J, Cotter T, Redmond H (2013). Less stress, more success? Oncological implications of surgery-induced oxidative stress. Gut.

[b53] Pekarek LA, Starr BA, Toledano AY, Schreiber H (1995). Inhibition of tumor growth by elimination of granulocytes. J Exp Med.

[b54] Petrie TA, Strand NS, Tsung-Yang C, Rabinowitz JS, Moon RT (2014). Macrophages modulate adult zebrafish tail fin regeneration. Development.

[b55] Redd MJ, Kelly G, Dunn G, Way M, Martin P (2006). Imaging macrophage chemotaxis in vivo: studies of microtubule function in zebrafish wound inflammation. Cell Motil Cytoskeleton.

[b56] Retsky M, Demicheli R, Hrushesky WJ, Forget P, De Kock M, Gukas I, Rogers RA, Baum M, Sukhatme V, Vaidya JS (2013). Reduction of breast cancer relapses with perioperative non-steroidal anti-inflammatory drugs: new findings and a review. Curr Med Chem.

[b57] Rhodes J, Hagen A, Hsu K, Deng M, Liu TX, Look AT, Kanki JP (2005). Interplay of pu.1 and gata1 determines myelo-erythroid progenitor cell fate in zebrafish. Dev Cell.

[b58] Riber-Hansen R, Hastrup N, Clemmensen O, Behrendt N, Klausen S, Ramsing M, Spaun E, Hamilton-Dutoit SJ, Steiniche T (2012). Treatment influencing down-staging in EORTC Melanoma Group sentinel node histological protocol compared with complete step-sectioning: a national multicentre study. Eur J Cancer.

[b59] Robert C, Ribas A, Wolchok JD, Hodi FS, Hamid O, Kefford R, Weber JS, Joshua AM, Hwu WJ, Gangadhar TC, Patnaik A, Dronca R, Zarour H, Joseph RW, Boasberg P, Chmielowski B, Mateus C, Postow MA, Gergich K, Elassaiss-Schaap J (2014). Anti-programmed-death-receptor-1 treatment with pembrolizumab in ipilimumab-refractory advanced melanoma: a randomised dose-comparison cohort of a phase 1 trial. Lancet.

[b60] Rothwell PM, Fowkes FGR, Belch JFF, Ogawa H, Warlow CP, Meade TW (2011). Effect of daily aspirin on long-term risk of death due to cancer: analysis of individual patient data from randomised trials. Lancet.

[b61] Rothwell PM, Price JF, Fowkes FGR, Zanchetti A, Roncaglioni MC, Tognoni G, Lee R, Belch JFF, Wilson M, Mehta Z, Meade TW (2012). Short-term effects of daily aspirin on cancer incidence, mortality, and non-vascular death: analysis of the time course of risks and benefits in 51 randomised controlled trials. Lancet.

[b62] Santoriello C, Gennaro E, Anelli V, Distel M, Kelly A, Koster RW, Hurlstone A, Mione M (2010). Kita driven expression of oncogenic hras leads to early onset and highly penetrant melanoma in zebrafish. PLoS ONE.

[b63] Schuh AC, Keating SJ, Monteclaro FS, Vogt PK, Breitman ML (1990). Obligatory wounding requirement for tumorigenesis in v-jun transgenic mice. Nature.

[b64] Senet P, Combemale P, Debure C, Baudot N, Machet L, Aout M, Vicaut E, Lok C (2012). Malignancy and chronic leg ulcers: the value of systematic wound biopsies: a prospective, multicenter, cross-sectional study. Arch Dermatol.

[b65] Sieweke MH, Stoker AW, Bissell MJ (1989). Evaluation of the Cocarcinogenic Effect of Wounding in Rous Sarcoma Virus Tumorigenesis. Cancer Res.

[b66] Sieweke MH, Thompson NL, Sporn M, Bissell M (1990). Mediation of wound-related Rous sarcoma virus tumorigenesis by TGF-b. Science.

[b67] Sionov RV, Fridlender ZG, Granot Z (2014). The multifaceted roles neutrophils play in the tumor microenvironment. Cancer Microenviron.

[b68] Topalian SL, Hodi FS, Brahmer JR, Gettinger SN, Smith DC, McDermott DF, Powderly JD, Carvajal RD, Sosman JA, Atkins MB, Leming PD, Spigel DR, Antonia SJ, Horn L, Drake CG, Pardoll DM, Chen L, Sharfman WH, Anders RA, Taube JM (2012). Safety, activity, and immune correlates of anti-PD-1 antibody in cancer. N Engl J Med.

[b69] Troester MA, Lee MH, Carter M, Fan C, Cowan DW, Perez ER, Pirone JR, Perou CM, Jerry DJ, Schneider SS (2009). Activation of host wound responses in breast cancer microenvironment. Clin Cancer Res.

[b70] Werner S, Schafer M (2008). Cancer as an overhealing wound: an old hypothesis revisited. Nat Rev Mol Cell Biol.

[b71] Westerfield M (2007). The Zebrafish Book: a Guide for the Laboratory Use of Zebrafish (Danio Rerio).

[b72] Wolchok JD, Kluger H, Callahan MK, Postow MA, Rizvi NA, Lesokhin AM, Segal NH, Ariyan CE, Gordon RA, Reed K, Burke MM, Caldwell A, Kronenberg SA, Agunwamba BU, Zhang X, Lowy I, Inzunza HD, Feely W, Horak CE, Hong Q (2013). Nivolumab plus ipilimumab in advanced melanoma. N Engl J Med.

[b73] Yang L, Qiu CX, Ludlow A, Ferguson MW, Brunner G (1999). Active transforming growth factor-beta in wound repair: determination using a new assay. Am J Pathol.

