# Supplementary figures and images for: The wound inflammatory response exacerbates growth of pre-neoplastic cells and progression to cancer
Source: EMBO J. 2015 Jul 1;34(17):2219–36. doi: 10.15252/embj.201490147 (PMC4585460; doi:10.15252/embj.201490147)

Figure S1

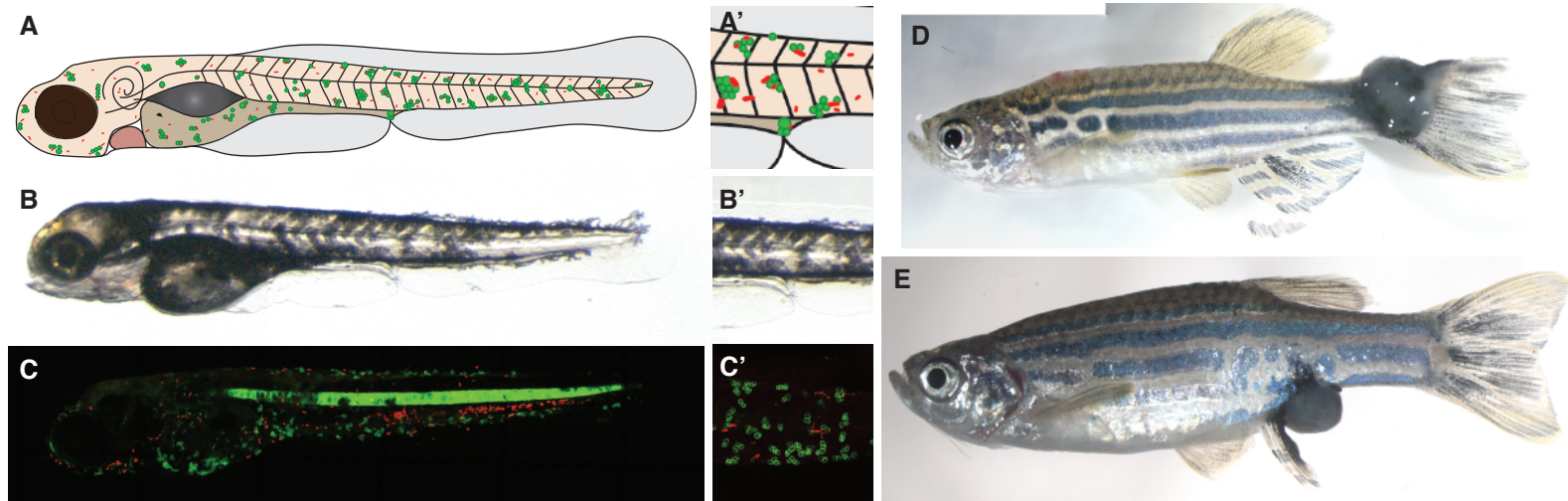

Supplement: Supplementary file 1 [file embj0034-2219-sd1.pdf]

Figure S2

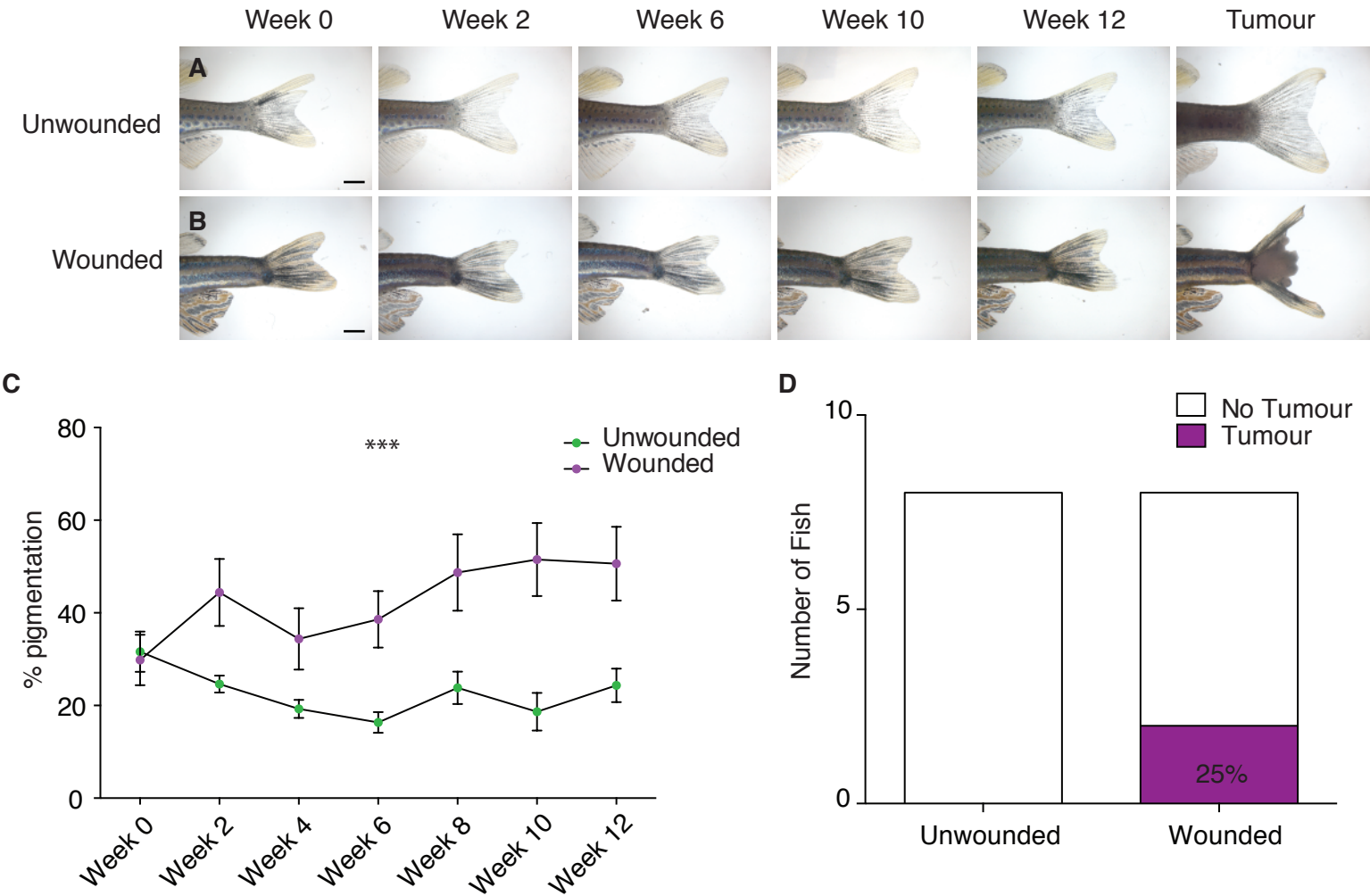

Supplement: Supplementary file 2 [file embj0034-2219-sd2.pdf]

Figure S3

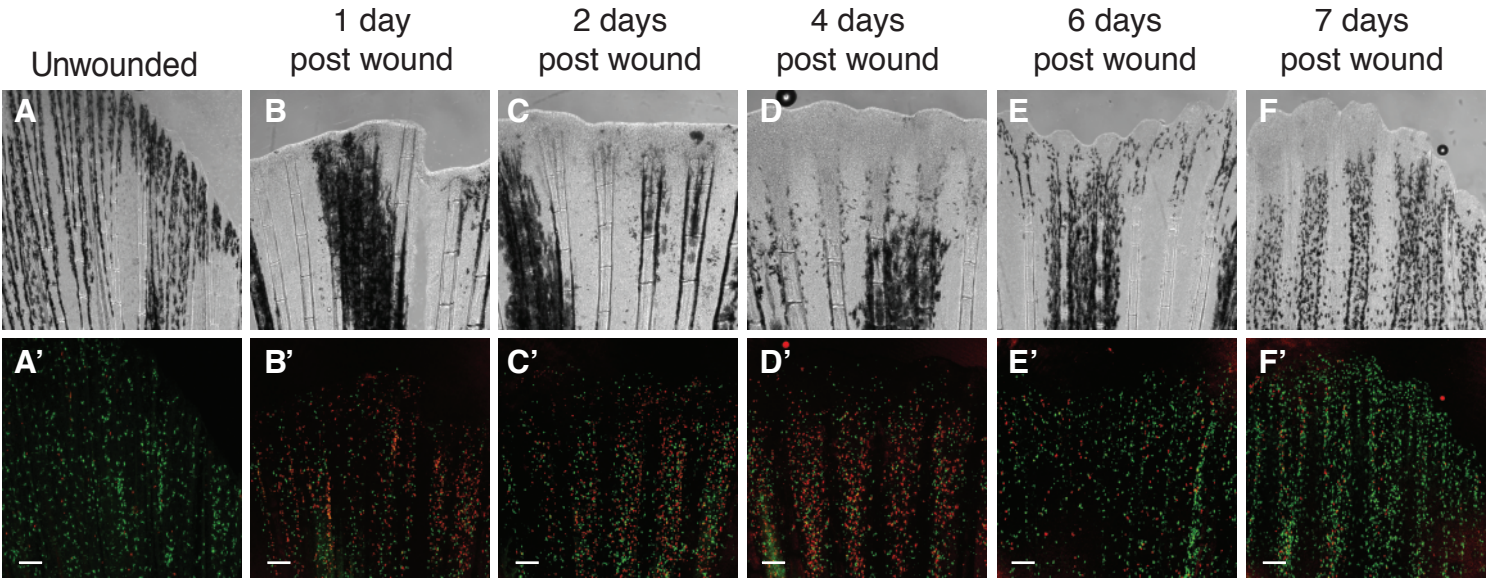

Two days post wound zoom

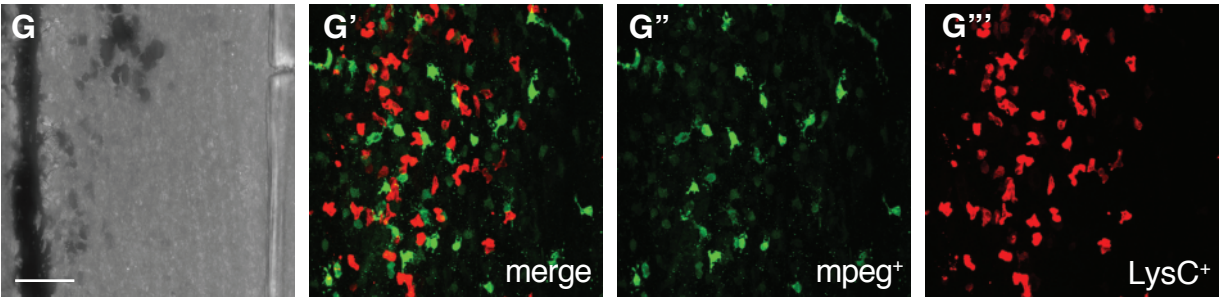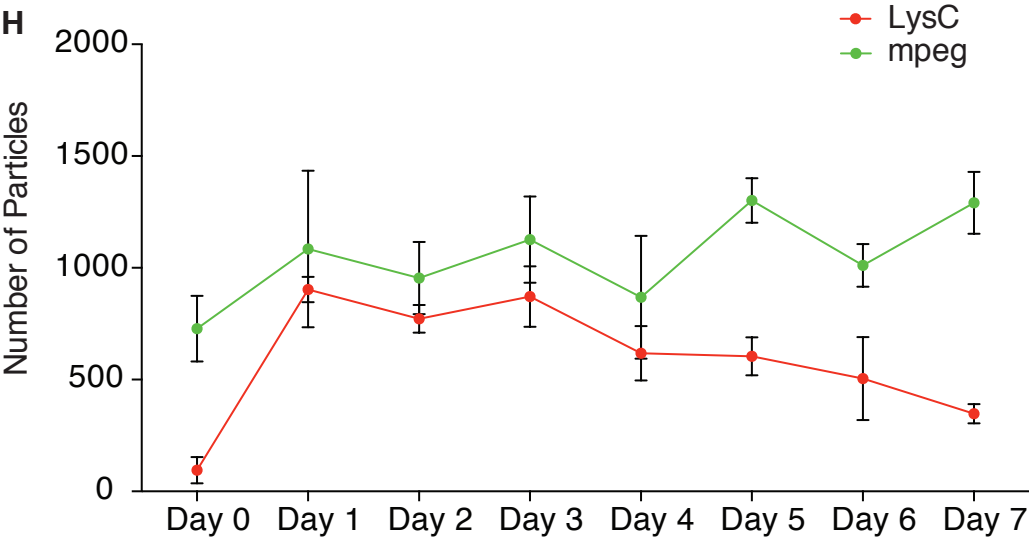

Supplement: Supplementary file 3 [file embj0034-2219-sd3.pdf]

Figure S4

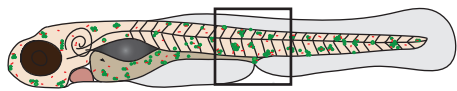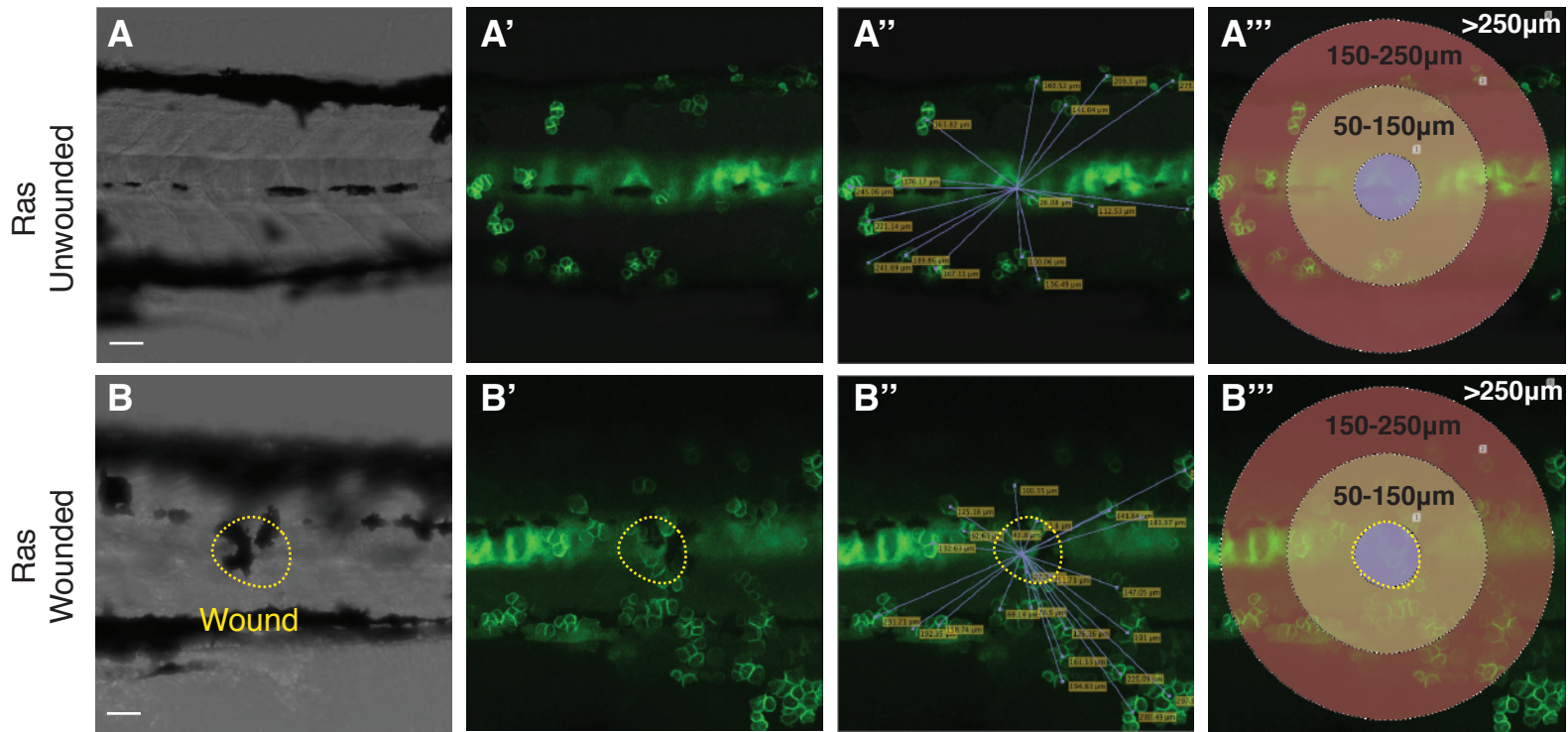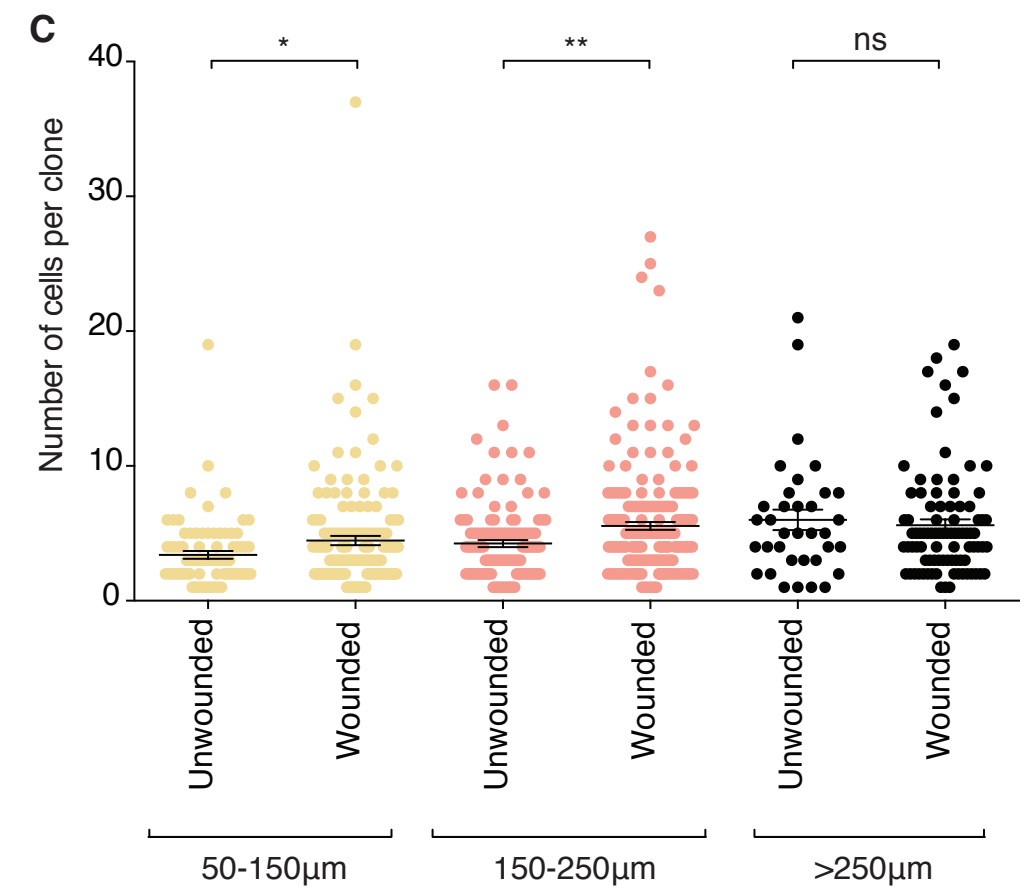

Supplement: Supplementary file 4 [file embj0034-2219-sd4.pdf]

Figure S5

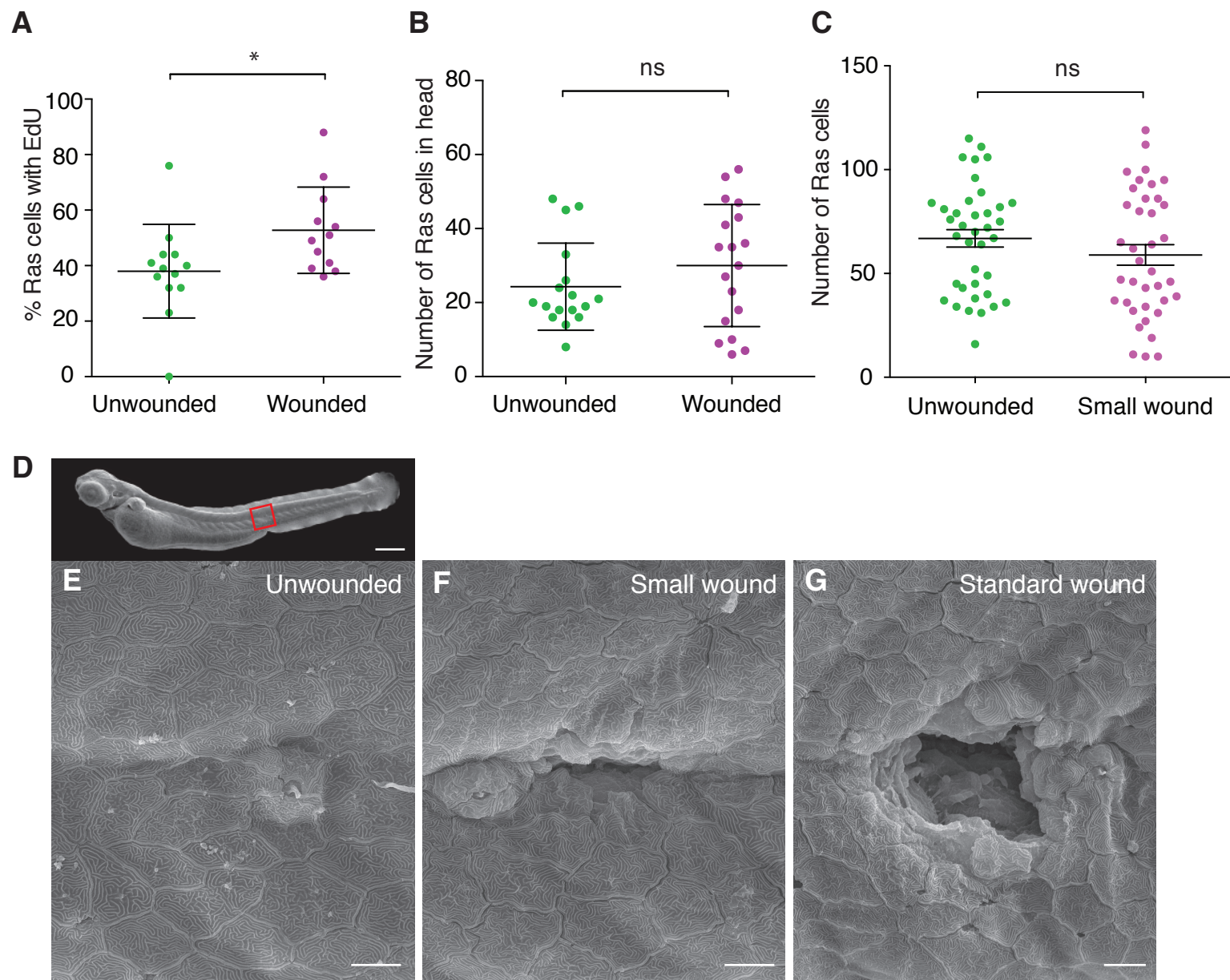

2dpf Ras<sup>+</sup> larvae

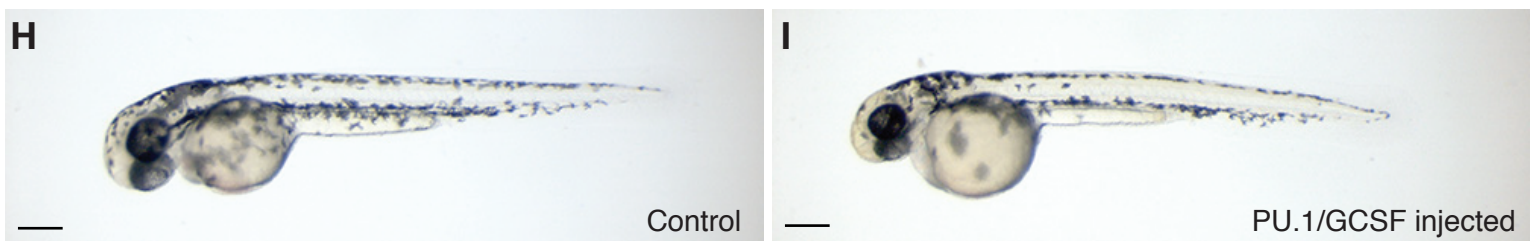

3dpf Ras<sup>+</sup> larvae

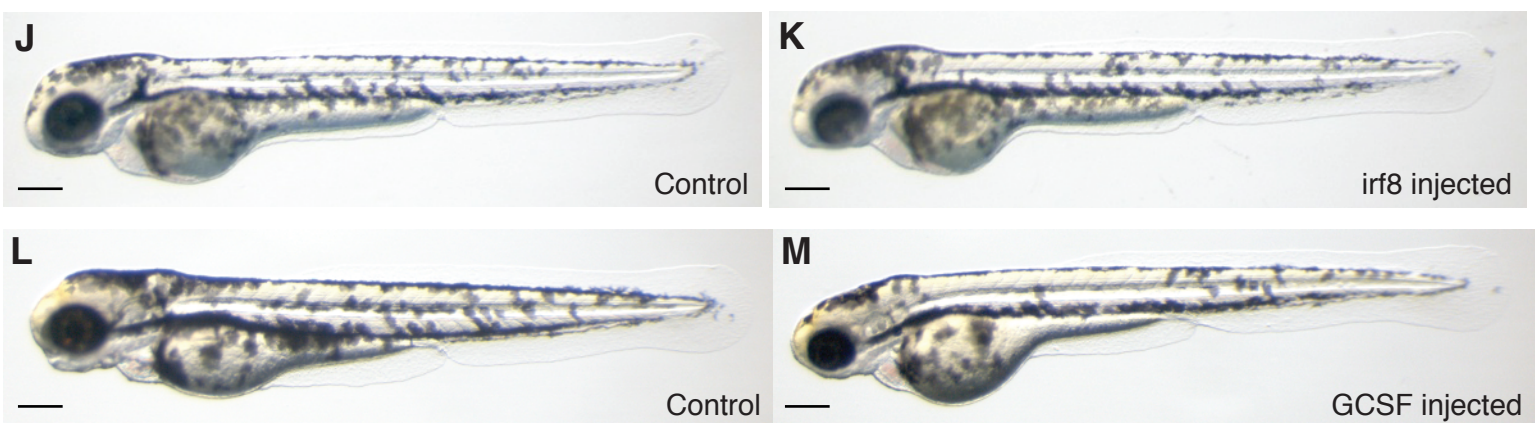

Supplement: Supplementary file 5 [file embj0034-2219-sd5.pdf]

Figure S6

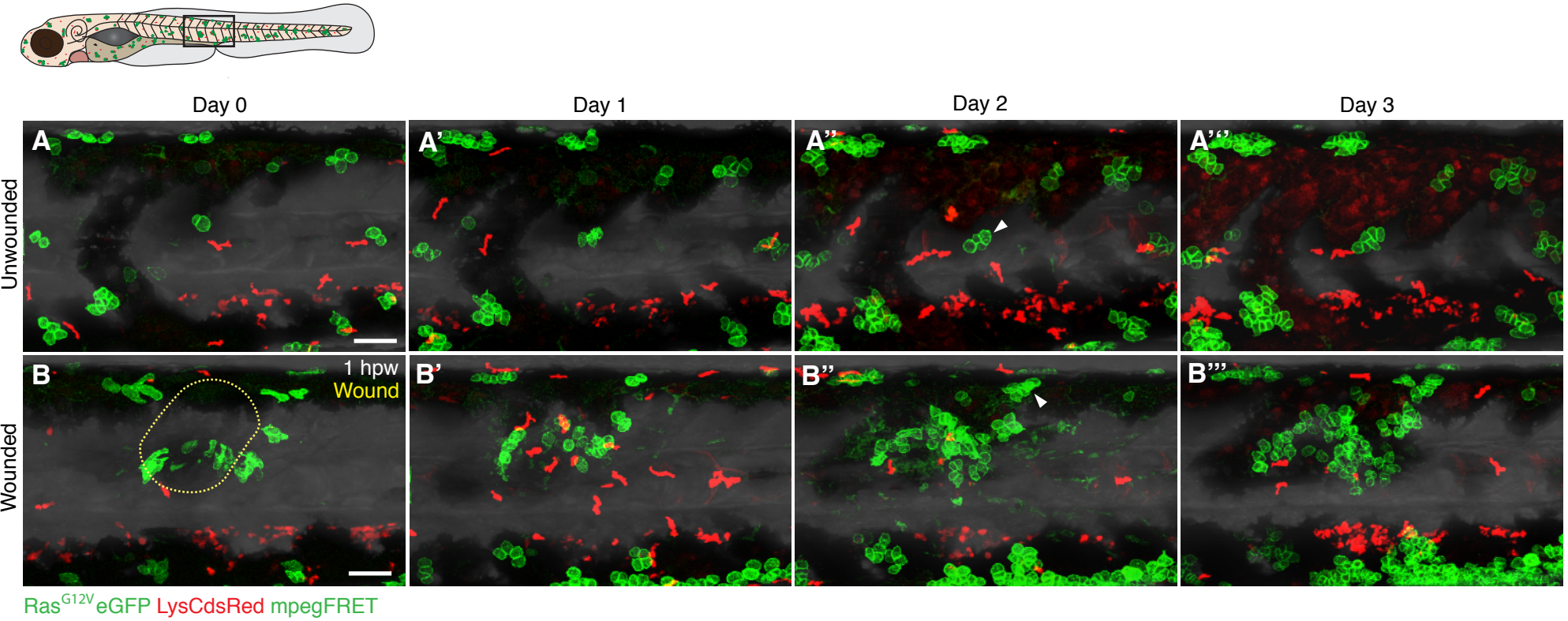

Supplement: Supplementary file 6 [file embj0034-2219-sd6.pdf]
