# Supplementary material for: The wound inflammatory response exacerbates growth of pre-neoplastic cells and progression to cancer
Source: EMBO J. 2015 Jul 1;34(17):2219–36. doi: 10.15252/embj.201490147 (PMC4585460; doi:10.15252/embj.201490147)
Supplement: Supplementary file 7 [file embj0034-2219-sd7.docx]

**Table S1 - Correlation between the extent of ulceration and tumour-cell proliferation, infiltration of neutrophils and infiltration of macrophages.**

|  |  | Estimate (95% CI) | P-value |
| --- | --- | --- | --- |
| Proliferation (Ki67 area fraction %) | No ulceration (ref.) |  |  |
|  | Minimal ulceration (<70%) | 1.70 (1.37-2.12) | p=0.0004 |
|  | Excessive ulceration (>70%) | 2.07 (1.43-2.98) | p=0.0001 |
| Macrophages (cd163+ area fraction %) | No ulceration (ref.) |  |  |
|  | Minimal ulceration (<70%) | 1.13 (0.69-1.86) | p=0.88 |
|  | Excessive ulceration (>70%) | 1.16 (0.56-250.00) | p=0.90 |
| Neutrophils (cd66b+ area fraction %) | No ulceration (ref.) |  |  |
|  | Minimal ulceration (<70%) | 15.02 (8.62-26.19) | p<0.0001 |
|  | Excessive ulceration (>70%) | 99.94 (39.22-254.64) | p<0.0001 |

**Table S2 - Correlation between tumour-cell proliferation and infiltration of neutrophils and infiltration of macrophages.**

|  | Proliferation (Ki67 area fraction %) | P-value |
| --- | --- | --- |
| Macrophages (cd163+ area fraction %) | No significant interactions | 0.56 |
| Neutrophils (cd66b+ area fraction %) | Significant interaction with ulceration | 0.0002 |

**Table S3 - Uni- and multivariate melanoma specific survival-analysis, adjusted for Breslow thickness, ulceration, sentinel node status and the case/control study design.**

|  |  | Univariate analysis |  | Multivariate analysis |  |
| --- | --- | --- | --- | --- | --- |
| Proliferation (Ki67 area fraction %) | n=336 | HR= 1.44 (1.19-1.76) | p=0.0002 | HR=1.19 (0.97-1.46) | p=0.10 |
|  |  |  |  |  |  |
| Macrophages (cd163+ area fraction %) | n=381 | HR= 1.03 (0.98-1.09) | p=0.24 | HR=1.02 (0.96-1.09) | p=0.44 |
|  |  |  |  |  |  |
| Neutrophils (cd66b+ area fraction %) | n=380 |  |  |  | p<0.0001 |
|  |  |  |  |  |  |
| No ulcerations |  | HR= 1.13 (1.05-1.21) | p=0.0007 | 1.10 (1.01-1.20) | p=0.02 |
| Minimal ulcerations |  | HR= 0.97 (0.89-1.05) | p=0.43 | 0.96 (0.88-1.04) | p=0.29 |
| Excessive ulcerations |  | HR= 1.23 (0.98-1.53) | p=0.077 | 1.34 (1.03-1.74) | p=0.03 |
