# Supplementary material for: The wound inflammatory response exacerbates growth of pre-neoplastic cells and progression to cancer
Source: EMBO J. 2015 Jul 1;34(17):2219–36. doi: 10.15252/embj.201490147 (PMC4585460; doi:10.15252/embj.201490147)
Supplement: Supplementary file 12 [file embj0034-2219-sd12.docx]

**Figure S1 - Standard larval model for studying immune cell:pre-neoplastic cell interactions.**

A Schematic to illustrate the larval model, whereby oncogenic Human Ras^G12V^ is used to drive cell transformation in a tissue specific manner, in melanocytes and mucus-secreting goblet cells by a *kit-a* enhancer trap (ET30).

B DIC microscopy of a 5dpf *kita*:Ras^G12V^eGFP zebrafish larvae.

C Confocal laser scanning microscopy image of a 5dpf *kita*:Ras^G12V^eGFP; LysC:dsRed zebrafish larvae. (A’, B’, C’) Zoom in of the area around the cloaca to illustrate the region frequently imaged.

D and E Adult fish occasionally develop large pigmented tumours, particularly at exposed sites such as the ventral fin by the cloaca or tail.

**Figure S2 - Wounding adult fish leads to tumour development.**

A Unwounded 3-6 month old *kita*:Ras^G12V^eGFP fish followed over time.

B 3-6 month old *kita*:Ras^G12V^eGFP fish wounded by tail-fin resection every 2 weeks for 12 weeks. Scale bars represent 2mm (A-B).

C Graph to illustrate the % pigmentation over 12 weeks.

D Number of tail fin tumours developed in wounded versus unwounded fish (n = 8 fish in unwounded and wounded group respectively).

**Figure S3 - Timecourse of leukocyte recruitment to adult fin wounds.**

A-F Adult zebrafish were analysed for inflammatory cell recruitment by resecting a small portion of the caudal tail fin. Three 6 month old LysC:dsRed; mpeg:FRET fish were fixed each day post wounding and whole-mount immuno-stained for LysC+ neutrophils (red) and mpeg+ macrophages (green) for seven days post wounding.

G-G’’’ A zoom in of immune cell recruitment at two days post wound (C) is shown in panel.

H- Neutrophil and macrophage recruitment is quantified, calculated by the number of particles counted automatically in ImageJ.

Scale bars represent 150μm (A-F and J-O) and 50μm (G).

**Figure S4 - Clone size increases in a zone extending up to 250μm from the wound centre.**

A-B RasG12VeGFP larvae were left unwounded (A) or laser wounded (B) at 2dpf just dorsal to the urogenital opening. Larvae were left to grow for 3 days before being fixed and analysed for pre-neoplastic cell number (n=30 larvae in each group). (A’’ and B’’) show the analysis performed to measure the distance from a clone to the centre of the wound. (A’’’ and B’’’) show the boundaries of, 50-150μm, 150-250μm and >250 μm which were used to define clone location, as plotted in (C). The approximate domain for imaging is indicated by a box in the schematic.

Scale bars represent 50μm.

**Figure S5 - Only large wounds trigger proliferation in nearby pre-neoplastic cells.**

A Graph showing the percentage of EdU positive pre-neoplastic cells in unwounded versus wounded larvae.

B The number of pre-neoplastic cells distant from the wound site (in the larval head) is unchanged between wounded and unwounded larvae.

C Graph to show that small wounds (as shown in the SEM image, G) do not lead to increased proliferation.

D shows a whole zebrafish larvae with a box in red to indicate where high magnification pictures were taken of an unwounded 3dpf larvae (E), a small wound (F) where ablation is restricted to just one epithelial cell (although adjacent cells may also be affected), and a standard sized wound (G) which causes broader tissue damage across a diameter of 5-7 cells (i.e. >35μm).

H-M Brightfield images of Ras^+^ larvae injected with control morpholino (H, J and L) PU-1/GCSF morpholino (I) irf8 morpholino (K) and GCSF morpholino (M) at 2 or 3dpf (labelled). Scale bars represent 200μm (D and H - M) and 10μm (E-G).

**Figure S6 - Timecourse of clonal growth in the presence versus absence of a wound.**

A-B A series of time-lapse images showing clonal growth in a wounded Ras^G12V^eGFP LysCdsRed mpegFRET larva and unwounded control over a period of four consecutive days. (A) Control, unwounded larva, versus (B) Larva wounded at 4dpf. Arrowhead in (B’’) highlights a clone close to the wound, which has increased in size from two to seven cells over a 24 hour period. Over the same period, most clones in the control larva grow more slowly (arrowhead in A’’). The approximate domain for imaging is indicated by a box in the schematic. Scale bars represent 100μm.

**Supplementary Movie S1**

Movie of an unwounded Ras^+^ 5dpf larva; green cells are Ras^G12V^ positive pre-neoplastic mucus-secreting cells; red cells are LysC:dsRed labeled neutrophils.

**Supplementary Movie S2**

Movie of a wounded Ras^-^ sibling, 5dpf larva; red cells are LysC:dsRed labeled neutrophils. The larva was wounded just above the cloaca by a laser, 90 minutes prior to imaging.

**Supplementary Movie S3**

Movie of a wounded Ras^+^ 5dpf larva; green cells are Ras^G12V^ positive pre-neoplastic mucus-secreting cells; red cells are LysC:dsRed labeled neutrophils. The larva was wounded just above the cloaca by a laser, 90 minutes prior to imaging.

**Supplementary Movie S4**

Movie of a wounded Ras^+^ 5dpf larva; green cells are Ras^G12V^ positive pre-neoplastic mucus-secreting cells; red cells are LysC:dsRed labeled neutrophils. The larva was treated with 100μM DPI inhibitor from 60 minutes prior to wounding, then wounded just above the cloaca by a laser, 90 minutes prior to imaging. Wound outline is indicated by a yellow circle.
