# Supplementary material for: The wound inflammatory response exacerbates growth of pre-neoplastic cells and progression to cancer
Source: EMBO J. 2015 Jul 1;34(17):2219–36. doi: 10.15252/embj.201490147 (PMC4585460; doi:10.15252/embj.201490147)
Supplement: Supplementary file 13 [file embj0034-2219-sd13.pdf]

THE EMBO JOURNAL

Manuscript EMBO-2014-90147

# The wound inflammatory response exacerbates growth of pre-neoplastic cells and progression to cancer

Nicole Antonio, Marie Louise Bønnelykke-Behrndtz, Laura Ward, John Collin, Ib Jarle Christensen, Torben Steiniche, Henrik Schmidt, Yi Feng and Paul Martin

*Corresponding author: Paul Martin, Bristol, University of -*

**Review timeline:**

| | |
|---|---|
| Submission date: | 30 September 2014 |
| Editorial Decision: | 04 November 2014 |
| Revision received: | 15 April 2015 |
| Editorial Decision: | 25 May 2015 |
| Accepted: | 25 May 2015 |

Editor: Karin Dumstrei

**Transaction Report:**

(Note: With the exception of the correction of typographical or spelling errors that could be a source of ambiguity, letters and reports are not edited. The original formatting of letters and referee reports may not be reflected in this compilation.)

1st Editorial Decision 04 November 2014

Thank you for submitting your manuscript to The EMBO Journal. Your study has now been seen by three referees and their comments are provided below.

As you can see, the referees find the analysis interesting, insightful and suitable for publication here. However, they also raise a number of issues that should be addressed. I anticipate that you should be able to address the concerns raised within a reasonable timeframe. There are some concerns raised regarding the PGE2 dataset. I don't expect you to sort out which cell type contributes most to providing PGE2. If you have data on hand to address this issue then please include it, but if not that is fine as well. I am happy to discuss the referee points further if needed.

When preparing your letter of response to the referees' comments, please bear in mind that this will form part of the Review Process File, and will therefore be available online to the community. For more details on our Transparent Editorial Process, please visit our website: http://emboj.embopress.org/about#Transparent_Process

Looking forward to seeing the revised manuscript

REFEREE REPORTS

Referee #1:

In this study Antonio and colleagues present a zebrafish model to study how wounds affect cancer progression.

The main conclusion of this paper is that neutrophils move from wounds towards Ras-pre-neoplastic cells and that these interactions lead to increased proliferation among Ras cells.

While this topic is surely interesting, data are rather hard to interpret and fall short in supporting main conclusions.

For example:

-The authors examine the response of inflammatory cells in adults in the presence of a wound. They claim that neutrophils are the first cells to appear 24hrs post injury, while macrophages are recruited more slowly. Thus, from the graph in the extended view 2K we conclude that neutrophils are represented by the red curve (marked by L-plastin), while macrophages are the yellow curve (marked by LysC). However, this is in contrast with Fig.1I where it is the opposite with macrophages been marked by L-plastin and neutrophils by LysC. This is also in contrast with images in the extended view 2E-J where LysC (yellow) seems to appear first.
In addition, the use of L-plastin to label macrophages exclusively and LysC for neutrophils is questionable. Indeed, in the fish larva these are both myeloid markers. Please, see Barman et al. Exp. Hematology 2005; Hall et al. BMC Dev.Biol. 2007 7:42 "lysC... labeled cells were shown by co-expression studies and FACS analysis to represent a subset of macrophages and likely also granulocytes". And also Kitaguchi et al. MOD 2009, where LysC is described as a marker for myeloid cells, thus both macrophages and neutrophils. This is a very important aspect that should be clarified. The figures do not allow distinguishing single cells. The authors should also provide higher magnifications.
In Fig.2 G-J how do the authors visualize LysC- cells? Moreover, how do they quantify macrophages in the graph in Fig.3Q?
In my view, the quality of Fig.1I-L is not sufficient to support the claim that "there is a clear concentration of neutrophils at the cut edge of the remaining cancer". This is not visible and there is no control image for comparison.

-In Fig. 2R the authors quantify the number of pre-neoplastic cells receiving contacts from immune cells. How did they define and quantify contacts? How long do these contacts last? What is the relevance of cell-cell contacts? Do Ras cells proliferate as a result of a direct contact?

-One important conclusion in this study is that in the presence of a wound, and of immune cells, there is increased proliferation among Ras neoplastic cells. This is shown in Fig.3 by quantifying the number of pre-neoplastic cells in the presence (A'-D') and absence (A-D) of a wound. I imagine Ras fish to vary a lot in the amount of Ras expressing cells, and for this reason the authors should show the same transgenic animal before and after wounding so that readers can get a better sense of the increase. For the same reason, the graph in Fig.3E should represent data out of several independent replicas of the same experiment.

-It is important to show the localized effect that the presence of immune cells exerts on Ras neoplastic cells proliferation and to determine the range of action that a wound has on the proliferation of surrounding cells. Can the range of movement of myeloid cells out of the wound be correlated with increased proliferation? Indeed, we would expect areas that are far for the wound to have normal proliferation rate. These data will also constitute a powerful internal control for these experiments.

Fig.3 A-D, A'-D', F-K and F'-K' lack labeling. What are red and pink stainings in these figures? Please check labeling of figures throughout the paper.

© European Molecular Biology Organization 

Referee #2:

By using a Zebrafish larval model of Ras-driven neoplasia, the manuscript by Nicole Antonio et al, entitled "The wound inflammatory response exacerbate growth of pre-neoplastic cells and progression to cancer", indicates that wounding promotes a rapid interaction between neutrophils and pre-neoplastic cells, leading to their increased proliferation. In addition, the authors indicate PGE2 as a signal expressed in sites of wound-inflammation and promoting neoplastic cell proliferation. The association between neutrophils and cancer cell proliferation was found also at sites of melanoma ulceration, which correlated with poor clinical outcome. The manuscript provides an elegant study to visualize the inflammatory events associating healing and cancer cell proliferation, which is of great relevance in cancer patients undergoing surgery, biopsy or ulceration.
Specific comments:
Fig. 3: by using morpholino against GCSF, the authors show that the delay of neutrophils development significantly depleted pre-neoplastic cell numbers, while inhibition of terminal maturation of macrophages obtained with a morpholino against IRF8 had only a partial effect on pre-neoplastic cells. They conclude that wound associated neutrophils are responsible for driving increased proliferation. Indeed, as the authors commented, macrophage depletion results in a compensatory increase in neutrophils, which may mask the macrophage contribution. Hence, despite the conclusion on neutrophils is realistic, the role of macrophages remain uncertain. In addition, the role of macrophage cannot be only correlated with their number, as these cells are known to express different polarized inflammatory programs during the onset and resolution of the inflammatory response, which are part of the healing process. The author should better discuss these aspects and their possible implications.

In Fig. 4 the authors indicate PGE2 as the trophic factor responsible for the wound-induced increase in proliferation of pre-neoplastic cells. Indeed, the rescue in the proliferative response of pre-neoplastic cells provided by PGE2 treatment is quite marginal, suggesting that PGE2 may be only a cofactor. In addition, it is not clear which cell type contributes most to the endogenous levels of PGE2. Thus this information remains partial.

Fig. 5 shows correlation between infiltration of neutrophils and advanced ulceration in melanoma. This is a convincing evidence. However, it looks like that increased macrophage infiltration occurs from "no ulceration" to "moderate ulceration" (panel D' and E') and, as compared to D', remains higher in "excessive ulceration" (panel F'). Can the authors clarify this evidence?

Overall, the manuscript provides clear indications supporting the role of would inflammatory neutrophils in growth of preneoplastic cells and progression to cancer. However, considering also the lack of evidence on the polarization status of macrophages, which is of obvious importance in cancer growth, the conclusions drawn on these cells can be somehow partial and would deserve proper wider investigations.

Referee #3:

In this manuscript the authors use a zebrafish wounding model to investigate interactions between the innate immune cell response and nearby neoplastic cells. They demonstrate that wounding enhances the proliferation of local cells containing oncogenic Ras. This effect is dependent upon neutrophils and is partly mediated by prostaglandins (although this part of the study is a little weak). In addition they observe a correlation between neutrophil presence in ulcerated melanoma and prognostic outcome in patients. Overall, I like the approach and think that this an interesting study. Nonetheless, there are some areas that need attention before it could be published.

Major comments

1. Is there a role for $H_2O_2$ in the process they describe? The authors previously described a role for peroxide in recruiting myeloid cells to pre-neoplastic cells (Feng et al PLoS Biology).

2. I am not convinced by the PGE2 data. First, NS398 does not block the induction of Ras cells by wounding - it reduces the baseline, but the fold change looks almost identical. Second, the addition of PGE2 has only a small ability to rescue the defects in PU1+GCSF depleted fish. Third, in the same experiment, wounding induces more Ras cells even when PGE2 levels are presumably saturating. Altogether, these data clearly indicate that there are other major factors involving in supporting the proliferation of Ras cells following myeloid cell infiltration. I interpret the data as indicating that PGE2 has a positive effect on Ras cells, but that PGE2 is not the dominant 'inductive' signal following wounding. The authors should investigate other Cox blocking compounds, perhaps Cox1&2 blockade (perhaps they are not hitting PGE2 synthesis hard enough in this experiment) would have a more impressive effect on blocking the induction of Ras cells following wounding, not just the baseline. If not, then the authors need to significantly moderate their language.

Minor comments

1. It is an over-statement to imply that the main link between HPV and cancer is through inflammation. It is well-established that HPV encodes viral oncogenes (E6 and E7) that play a major cell intrinsic role in the transformation of cervical cancer.

2. Figure order 3 should be rearranged so they figures are in the same order as mentioned in the text. In legend or figure should be added what red, green and/or yellow is. Also figure 3L is never mentioned in the text.

3. I am not convinced that the proportion of Edu+ve Ras cells is different between wounded and unwounded fish - in Figure 3D I count 3/5 +ve cells and in Figure 3D' I count 5/15 cells. Please clarify.

4. Would be interesting to quantify the increase in EdU positive pre-neoplastic cells as well as all other EdU positive cells in 2 days post wounding as a function of distance from the wound.

5. Figure 5: add $R^2$ and p-value in the graph to aid readability

6. The authors talk about proliferation after wounding, but also clinical correlations are made regarding survival, mets? Are there any indications that neutrophils make melanoma cells more motile/invasive?

7. The authors should cite relevant literature from the Watt group - Arwert et al PNAS 2010 and Arwert et al Oncogene 2012.

8. I would like some discussion about whether the effects depend on the oncogene expressed in the pre-neoplastic cells. Would neutrophils have a similar effect on pre-neoplastic cells with an oncogenic β-catenin mutation?

1st Revision - authors' response 15 April 2015

© European Molecular Biology Organization 

Referee #1:

In this study Antonio and colleagues present a zebafish model to study how wounds affect cancer progression.
The main conclusion of this paper is that neutrophils move from wounds towards Ras-pre-neoplastic cells and that these interactions lead to increased proliferation among Ras cells.

While this topic is surely interesting, data are rather hard to interpret and fall short

in supporting main conclusions.

For example:

-The authors examine the response of inflammatory cells in adults in the presence of a wound. They claim that neutrophils are the first cells to appear 24hrs post injury, while macrophages are recruited more slowly. Thus, from the graph in the extended view 2K we conclude that neutrophils are represented by the red curve (marked by L-plastin), while macrophages are the yellow curve (marked by LysC). However, this is in contrast with Fig.1I where it is the opposite with macrophages been marked by L-plastin and neutrophils by LysC. This is also in contrast with images in the extended view 2E-J where LysC (yellow) seems to appear first.
In addition, the use of L-plastin to label macrophages exclusively and LysC for neutrophils is questionable. Indeed, in the fish larva these are both myeloid markers. Please, see Barman et al. Exp. Hematology 2005; Hall et al. BMC Dev.Biol. 2007 7:42 "lysC... labeled cells were shown by co-expression studies and FACS analysis to represent a subset of macrophages and likely also granulocytes". And also Kitaguchi et al. MOD 2009, where LysC is described as a marker for myeloid cells, thus both macrophages and neutrophils. This is a very important aspect that should be clarified.
The figures do not allow distinguishing single cells. The authors should also provide higher magnifications.

**Sorry for the confusion here, and we hope we have clarified things now. Part of the confusion was because our L-plastin antibody, which is leukocyte specific in larvae, reveals all white cell lineages, including adaptive immune cells, plus some additional cells, in the adult. We have now wounded the tail of adult LysC:dsRed; mpeg:CFP-YFP fish (a newly available line) to more clearly distinguish adult macrophages and neutrophils. These new data are illustrated in extended view 3. We have also included high resolution images to reveal individual cell morphology (Extended View 3G).**

**This data also mirrors the immune cell recruitment found in Ras+ zebrafish in our original submission, where identifying macrophage populations, specifically, had not been possible but neutrophils (LysC+) were observed to peak at one day post tail fin amputation.**

In Fig.2 G-J how do the authors visualize LysC- cells? Moreover, how do they quantify macrophages in the graph in Fig.3Q?

**We presume the referee is referring to 2Q as there is no 3Q. To measure larval immune cell recruitment, LysCdsRed positive larvae were**

**immunostained for L-plastin at various timepoints post injury. All LysC negative, L-plastin positive cells (shown in red in Figure G-J) were presumed to be macrophages, and these were counted manually to produce the graph in Figure 2Q. We have clarified this in the Methods section pp 19 and added labels to the images.**

In my view, the quality of Fig.1I-L is not sufficient to support the claim that "there is a clear concentration of neutrophils at the cut edge of the remaining cancer". This is not visible and there is no control image for comparison.

**We have now added additional figures and a new model to help clarify this. Fig 1G is the "0 hr" wounded tumour imaged for neutrophils after resection from the fish. In I we show neutrophil accumulation at what had been the cut site of the tumour. To better illustrate this John Collin in the lab (a new author on the MS) has developed another cancer wound assay involving a punch biopsy through flatter melanomas, selected earlier, on the tail fin that are easier to image in the live fish. Here we show clear neutrophil recruitment to both wounded healthy and tumour tissue at 24 hrs post-biopsy but later, at three days, the neutrophils have resolved from the healthy wound but are retained in the tumour tissue (Fig 1K-M). As well as the new images in Fig 1, we have reworded the text of both Results (page 5) and Methods (page 20) sections to accommodate these new experiments.**

-In Fig. 2R the authors quantify the number of pre-neoplastic cells receiving contacts from immune cells. How did they define and quantify contacts? How long do these contacts last? What is the relevance of cell-cell contacts? Do Ras cells proliferate as a result of a direct contact?

**We used observed contacts as a proxy for immune cell:preneoplastic cell interactions; these contacts ranged from less than one minute to the whole duration of the movie (3 hours) and were quantified manually from many videos. We have added some more information about contact times etc in the text (pp 7) but we have no evidence that contacts, per se, are necessary for the local immune cell influences over pre-neoplastic cell growth that we observe (although we are currently developing strategies that might enable us to test this in the future) and so we have now added a sentence (pp 7) to avoid this possible impression.**

-One important conclusion in this study is that in the presence of a wound, and of immune cells, there is increased proliferation among Ras neoplastic cells. This is shown in Fig.3 by quantifying the number of pre-neoplastic cells in the presence (A'-D') and absence (A-D) of a wound. I imagine Ras fish to vary a lot in the amount of Ras expressing cells, and for this reason the authors should show the

same transgenic animal before and after wounding so that readers can get a better sense of the increase. For the same reason, the graph in Fig.3E should represent data out of several independent replicas of the same experiment.

**The reviewer is right; there is some variability in numbers of pre-neoplastic clones per fish. We now include, in our extended view 6, a timelapse series of a typical fish at the time of wounding and for three days subsequently, versus a control unwounded fish to show how clone size is increased in the proximity of a wound; this is reported in the text, pp 9. As for collecting several independent replicas of the same experiment, this is already the case; for Fig 3E there were three independent experiments. We have now addressed this in the legend to this figure.**

-It is important to show the localized effect that the presence of immune cells exerts on Ras neoplastic cells proliferation and to determine the range of action that a wound has on the proliferation of surrounding cells. Can the range of movement of myeloid cells out of the wound be correlated with increased proliferation? Indeed, we would expect areas that are far for the wound to have normal proliferation rate. These data will also constitute a powerful internal control for these experiments.

**We agree. In the initial submission we reported that this proliferative effect on pre-neoplastic cells is not systemic since we don't see increase in size of clones on the head or yolk sac after flank wounding. We have now counted clone number in zones extending 50-150, 150-250 and greater than 250um from the wound centre and, indeed, we see a local tailing off of the proliferative surge that correlates with the furthest extent of immune cell emigration away from the wound, i.e. approx. 250um (see extended view 4). We mention this new experiment and analysis in the text, pp 8.**

Fig.3 A-D, A'-D', F-K and F'-K' lack labeling. What are red and pink stainings in these figures? Please check labeling of figures throughout the paper.

**Throughout Figure 3, pre-neoplastic cells are labeled in green, LysC+ neutrophils in yellow and LysC-; L-plastin+ macrophages in red. In Figure 3D and D' the EdU marker of proliferation is indicated in magenta/purple. We have now included these labels in the figure.**

Referee #2:

By using a Zebrafish larval model of Ras-driven neoplasia, the manuscript by Nicole Antonio et al, entitled "The wound inflammatory response exacerbate growth of pre-neoplastic cells and progression to cancer", indicates that wounding promotes a rapid interaction between neutrophils and pre-neoplastic

cells, leading to their increased proliferation. In addition, the authors indicate PGE2 as a signal expressed in sites of wound-inflammation and promoting neoplastic cell proliferation. The association between neutrophils and cancer cell proliferation was found also at sites of melanoma ulceration, which correlated with poor clinical outcome. The manuscript provides an elegant study to visualize the inflammatory events associating healing and cancer cell proliferation, which is of great relevance in cancer patients undergoing surgery, biopsy or ulceration.
Specific comments:
Fig. 3: by using morpholino against GCSF, the authors show that the delay of neutrophils development significantly depleted pre-neoplastic cell numbers, while inhibition of terminal maturation of macrophages obtained with a morpholino against IRF8 had only a partial effect on pre-neoplastic cells. They conclude that wound associated neutrophils are responsible for driving increased proliferation. Indeed, as the authors commented, macrophage depletion results in a compensatory increase in neutrophils, which may mask the macrophage contribution. Hence, despite the conclusion on neutrophils is realistic, the role of macrophages remain uncertain. In addition, the role of macrophage cannot be only correlated with their number, as these cells are known to express different polarized inflammatory programs during the onset and resolution of the inflammatory response, which are part of the healing process. The author should better discuss these aspects and their possible implications.

**True. It is the case that macrophages could also be playing a role, but it appears that neutrophils definitely are. Both macrophages and neutrophils can exist in different phenotypic states but this is currently difficult to address in zebrafish because we have few definitive markers for the M1/M2 and N1/N2 states. As we suspect the reviewer will agree, the extent to which these markers truly reflect phenotypic state is even controversial in mouse and man; we have extended our discussion of this issue (pp 13 and 14) and make clear the degree of uncertainty we have in a role for macrophages in the cancer/wound scenario.**

In Fig. 4 the authors indicate PGE2 as the trophic factor responsible for the wound-induced increase in proliferation of pre-neoplastic cells. Indeed, the rescue in the proliferative response of pre-neoplastic cells provided by PGE2 treatment is quite marginal, suggesting that PGE2 may be only a cofactor. In addition, it is not clear which cell type contributes most to the endogenous levels of PGE2. Thus this information remains partial

**Yes, we think it is only one of the trophic factors derived from Innate immune cells that leads to pre-neoplastic proliferation, hence the incomplete rescue of proliferation by PGE2 treatment. We, just as this referee, think our data suggest that there might be other more significant trophic signals. We have added text to make this more clear (pp 10).**

Fig. 5 shows correlation between infiltration of neutrophils and advanced ulceration in melanoma. This is a convincing evidence. However, it looks like that increased macrophage infiltration occurs from "no ulceration" to "moderate ulceration" (panel D' and E') and, as compared to D', remains higher in "excessive ulceration" (panel F'). Can the authors clarify this evidence?

**Yes, we see what the Referee means, but the statistical evidence from many sections showed no significant correlation between macrophage numbers and the extent of ulceration. Whilst TAMs are present in non-ulcerated melanomas, there generally appear to be very few neutrophils present in these clinical melanomas unless the tissue is wounded/ulcerated.**

Overall, the manuscript provides clear indications supporting the role of would inflammatory neutrophils in growth of preneoplastic cells and progression to cancer. However, considering also the lack of evidence on the polarization status of macrophages, which is of obvious importance in cancer growth, the conclusions drawn on these cells can be somehow partial and would deserve proper wider investigations.

**Of course the role of macrophage (and neutrophil) polarisation status is on our list of future investigations; we are planning novel means to isolate leukocytes that have contacted pre-neoplastic cells (this might also allow us to address the "cell contact" issue raised by referee 1) to begin asking how interactions alter the transcriptome and possibly also epigenome of immune cells, but these data are a way off, and beyond the scope of the current paper. Rather, we have extended our discussion to highlight the importance of this issue on pp 14.**

Referee #3:

In this manuscript the authors use a zebrafish wounding model to investigate interactions between the innate immune cell response and nearby neoplastic cells. They demonstrate that wounding enhances the proliferation of local cells containing oncogenic Ras. This effect is dependent upon neutrophils and is partly mediated by prostaglandins (although this part of the study is a little weak). In addition they observe a correlation between neutrophil presence in ulcerated melanoma and prognostic outcome in patients. Overall, I like the approach and think that this an interesting study. Nonetheless, there are some areas that need attention before it could be published.

Major comments

1. Is there a role for H2O2 in the process they describe? The authors previously described a role for peroxide in recruiting myeloid cells to pre-neoplastic cells (Feng et al PLoS Biology).

**You won't be surprised to hear that we presumed there would be a role for H2O2 in this process because Niethammer and colleagues (2009) showed it is a major recruitment signal to wounds and we followed up with our Plos Biol paper (Feng et al., 2010) to say that pre-neoplastic cells also recruit with H2O2. We have now treated wounded larvae with a pre-neoplastic burden with DPI (which blocks H2O2 synthesis) and this dramatically reduces the numbers of neutrophils drawn to wounds and consequently many fewer preneoplastic cells in the wound vicinity experience contacts with these innate immune cells. We have added this new data to the bar chart in Fig 2E and include a movie (Expanded View Movie 4).**

2. I am not convinced by the PGE2 data. First, NS398 does not block the induction of Ras cells by wounding - it reduces the baseline, but the fold change looks almost identical. Second, the addition of PGE2 has only a small ability to rescue the defects in PU1+GCSF depleted fish. Third, in the same experiment, wounding induces more Ras cells even when PGE2 levels are presumably saturating. Altogether, these data clearly indicate that there are other major factors involving in supporting the proliferation of Ras cells following myeloid cell infiltration. I interpret the data as indicating that PGE2 has a positive effect on Ras cells, but that PGE2 is not the dominant 'inductive' signal following wounding. The authors should investigate other Cox blocking compounds, perhaps Cox1&2 blockade (perhaps they are not hitting PGE2 synthesis hard enough in this experiment) would have a more impressive effect on blocking the induction of Ras cells following wounding, not just the baseline.
If not, then the authors need to significantly moderate their language.

**Much as we responded to Referee 2, we don't want to argue that PGE2 is even the predominant trophic signal; rather, it appears to be ONE of the trophic signals. We liked your suggestion to try other Cox blocking agents and to that end we have used a combination of NS398 and SC560 and also aspirin, which serves both as a cox 1 and 2 dual blocker and is also clinically relevant because of its known effects on reducing cancer incidence and metastasis, particularly of carcinomas and adenomas in the gut. However, we see no enhanced effect beyond what we we already reported in our first submission. This confirms our suspicion (and that of this Referee) that prostaglandins are only part players. We are now more explicit in the Discussion section, pp 16.**

Minor comments

1. It is an over-statement to imply that the main link between HPV and cancer is through inflammation. It is well-established that HPV encodes viral oncogenes (E6 and E7) that play a major cell intrinsic role in the transformation of cervical cancer.

**Yes, agreed, this statement was too simplistic and we have replaced this with a more clear cut liver cancer reference (**pp 2).

2. Figure order 3 should be rearranged so they figures are in the same order as mentioned in the text. In legend or figure should be added what red, green and/or yellow is. Also figure 3L is never mentioned in the text.

**We have now rearranged our text (pp 8) to better align with the figures.**

3. I am not convinced that the proportion of Edu+ve Ras cells is different between wounded and unwounded fish - in Figure 3D I count 3/5 +ve cells and in Figure 3D' I count 5/15 cells. Please clarify.

**We had counted this specimen differently but more importantly, these were only representative specimens, and 30 larvae of each group were included in this quantification, which revealed a significant difference. We have now included a pair of high magnification Edu images (Figs 3D and D'), that more appropriately reflect the increased proliferative index in our wounded versus unwounded specimens.**

4. Would be interesting to quantify the increase in EdU positive pre-neoplastic cells as well as all other EdU positive cells in 2 days post wounding as a function of distance from the wound.

**As we discuss in our response to Referee 1, we also were interested in the "range" of influence of the wound inflammatory response; this we have examined by counting clone size within zones extending out from the wound, and we find a tailing off of increased clone size, that approximately coincides with the range of the wound inflammatory effect (extended view 4).**

5. Figure 5: add R2 and p-value in the graph to aid readability
**We have now added this statistical information to the relevant figure and legends.**

6. The authors talk about proliferation after wounding, but also clinical correlations are made regarding survival, mets? Are there any indications that neutrophils make melanoma cells more motile/invasive?

**Good question. There was a recent paper in Nature, Bald et al, 2014, showing how irradiation of melanoma leads to neutrophil recruitment and this is associated with subsequent metastisis. We have now mentioned this paper in our Discussion (pp 14)**

7. The authors should cite relevant literature from the Watt group - Arwert et al PNAS 2010 and Arwert et al Oncogene 2012.

**We did cite Awert (PNAS 2010) in our initial submission, and have now added a further line of text (pp 13), to include their most recent paper Hoste et al (2015), which came out after our initial submission of this MS, and which shows a nice link between wound size and tumour incidence in a mouse.**

8. I would like some discussion about whether the effects depend on the oncogene expressed in the pre-neoplastic cells. Would neutrophils have a similar effect on pre-neoplastic cells with an oncogenic $\beta$-catenin mutation?

**Good question and one that we are considering addressing in future experiments, particularly when we begin to drive oncogenes specifically in the gut epithelium of zebrafish in order to better model colon cancer. We have added a line in our Discussion (page 17), to suggest that looking at lesions beyond V12Ras will be an important next step.**

Editorial Decision 25 May 2015

Thank you for submitting your revised manuscript to The EMBO Journal. Your manuscript has now been re-reviewed by referees #1 and 3. I am sorry for the slight delay in getting back to you with a decision, but I have now heard back from the referees.

As you can see below, both referees appreciate the introduced changes and support publication here. There are no further changes needed. I am therefore very please to accept the paper for publication here.

REFEREE REPORTS

Referee #1:

The authors have done a very good job in addressing my main concerns and they have provided additional data to support major findings. I believe this work to be of high interest and quality.

Referee #3:

The authors have responded appropriately to my original comments. It is a shame that the PGE2 data are not so impressive, however the authors' claims in the text are now in line with the data. Overall, this is very interesting work that should be published in EMBO J.

© European Molecular Biology Organization
